# Synaptic alterations in visual cortex reshape contrast-dependent gamma oscillations and inhibition-excitation ratio in a genetic mouse model of migraine

**DOI:** 10.1186/s10194-022-01495-9

**Published:** 2022-09-29

**Authors:** Nicolò Meneghetti, Chiara Cerri, Eleonora Vannini, Elena Tantillo, Angelita Tottene, Daniela Pietrobon, Matteo Caleo, Alberto Mazzoni

**Affiliations:** 1 The Biorobotics Institute, Scuola Superiore Sant’Anna, 56025 Pisa, Italy; 2 Department of Excellence for Robotics and AI, Scuola Superiore Sant’Anna, 56025 Pisa, Italy; 3 Neuroscience Institute, National Research Council (CNR), 56124 Pisa, Italy; 4 Fondazione Umberto Veronesi, 20122 Milan, Italy; 5 Department of Pharmacy, University of Pisa, 56126 Pisa, Italy; 6 Fondazione Pisana per la Scienza Onlus (FPS), 56017 Pisa, Italy; 7 Scuola Normale Superiore, 56100 Pisa, Italy; 8 Department of Biomedical Sciences, University of Padova, 35131 Padova, Italy; 9 Padova Neuroscience Center, University of Padova, 35131 Padova, Italy; 10 CNR Institute of Neuroscience, 35131 Padova, Italy

**Keywords:** Migraine, Visual cortex, Mice, Gamma oscillations, Spiking neurons networks, Familial-hemiplegic-type1-migraine, Mutual information

## Abstract

**Background:**

Migraine affects a significant fraction of the world population, yet its etiology is not completely understood. In vitro results highlighted thalamocortical and intra-cortical glutamatergic synaptic gain-of-function associated with a monogenic form of migraine (familial-hemiplegic-migraine-type-1: FHM1). However, how these alterations reverberate on cortical activity remains unclear. As altered responsivity to visual stimuli and abnormal processing of visual sensory information are common hallmarks of migraine, herein we investigated the effects of FHM1-driven synaptic alterations in the visual cortex of awake mice.

**Methods:**

We recorded extracellular field potentials from the primary visual cortex (V1) of head-fixed awake FHM1 knock-in (*n* = 12) and wild type (*n* = 12) mice in response to square-wave gratings with different visual contrasts. Additionally, we reproduced in silico the obtained experimental results with a novel spiking neurons network model of mouse V1, by implementing in the model both the synaptic alterations characterizing the FHM1 genetic mouse model adopted.

**Results:**

FHM1 mice displayed similar amplitude but slower temporal evolution of visual evoked potentials. Visual contrast stimuli induced a lower increase of multi-unit activity in FHM1 mice, while the amount of information content about contrast level remained, however, similar to WT.

Spectral analysis of the local field potentials revealed an increase in the β/low γ range of WT mice following the abrupt reversal of contrast gratings. Such frequency range transitioned to the high γ range in FHM1 mice. Despite this change in the encoding channel, these oscillations preserved the amount of information conveyed about visual contrast. The computational model showed how these network effects may arise from a combination of changes in thalamocortical and intra-cortical synaptic transmission, with the former inducing a lower cortical activity and the latter inducing the higher frequencies ɣ oscillations.

**Conclusions:**

Contrast-driven ɣ modulation in V1 activity occurs at a much higher frequency in FHM1. This is likely to play a role in the altered processing of visual information. Computational studies suggest that this shift is specifically due to enhanced cortical excitatory transmission. Our network model can help to shed light on the relationship between cellular and network levels of migraine neural alterations.

**Graphical Abstract:**

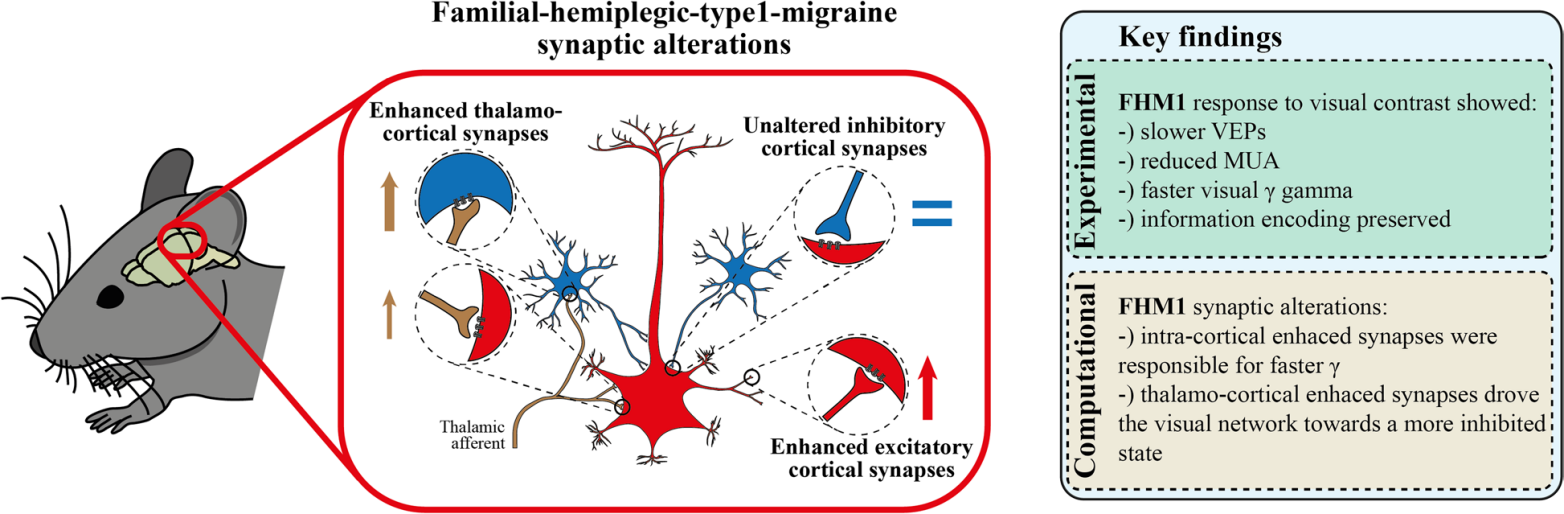

**Supplementary Information:**

The online version contains supplementary material available at 10.1186/s10194-022-01495-9.

## Background

Migraine is a complex disorder highly prevalent worldwide [1] and associated with a dysfunction in multisensory information processing. It usually manifests as recurrent episodes of unilateral headache, often anticipated by neurological symptoms, most frequently visual [2, 3]. Altered responsivity and abnormal processing of visual sensory information are common hallmarks of migraine. Aberrant integration and processing of incoming sensory information support the view of migraine as a neurological disorder characterized by a dysfunctional regulation of the balance between inhibition and excitation (I/E balance) within neuronal circuits of the cerebral cortex [4–6]. However, whether the dysfunctional sensory-evoked cortical activity is due to increased or decreased excitability of the primary visual cortex (V1) is still unclear [7–9].

Many genetic mouse models of monogenic subtypes of migraine have been engineered [10]. Here we adopted a genetic mouse model of pure familial hemiplegic migraine type 1 (FHM1) (van den Maagdenberg et al., 2004), which is a rare monogenic autosomal dominant form of migraine with aura [11]. FHM1 mutations result in gain-of-function of recombinant human Ca_V_2.1 channels and native neuronal Ca_V_2.1 channels in FHM1 knock-in mice, causing the enhancement of action potential evoked Ca^2+^ influx [12–15]. The FHM1 mouse model is consequently characterized by increased neurotransmission at both intra-cortical [15] and thalamocortical (TC) excitatory synapses [16]. FHM1 has a differential effect on short-term depression (STD) at TC synapses: compared to wild type (WT) mice, STD is greater at synapses contacting layer IV (L4) excitatory neurons while it is unaltered at synapses contacting L4 inhibitory neurons. As a result, during repetitive thalamic firing, the gain-of-function of TC excitatory inputs on inhibitory neurons becomes larger than that on excitatory neurons and the I/E balance is relatively skewed towards inhibition in FHM1 L4 excitatory neurons [16]. Inhibitory GABAergic transmission at different cortical inhibitory synapses remains instead unaltered in FHM1 despite being initiated by Ca_V_2.1 channels [15, 17].

Here we investigate the link between these synaptic alterations, I/E balance, and visual information processing by comparing neural activity recorded in V1 of awake WT and FHM1 mice in response to contrast reversal stimuli. We further present a spiking network model of FHM1 mice V1 able not only to reproduce experimental results but also to propose candidate mechanisms for the individual effects of each synaptic modification on the overall network activity.

## Methods

### Experimental model and subject details

#### Mice

All experimental procedures involving animals and their care were performed in accordance with National laws and policies (D.L. 26, March 14, 2014) and with the guidelines established by the European Community Council Directive (2010/63/UE) and were approved by the local authority veterinary services.

Animals were housed in a 12 h light/dark cycle with food and water available ad libitum. Experiments were performed using adult (4–6 weeks old) female C57BL6J wild-type (WT) mice and female homozygous knock-in mice carrying the Ca_V_2.1 R192Q FHM1 mutation with the same genetic background [12] with a medium weight of 25 g. The number of animals used in experiments in which the contrast level was increased up to 50 was *n* = 24 (*n* = 12 WT; *n* = 12 FHM1); the maximum contrast level (K = 90) was measured in only *n* = 14 (*n* = 7 WT; *n* = 7 FHM1) of these animals.

### Methods details

#### Visual stimuli

**Fig. 1:**
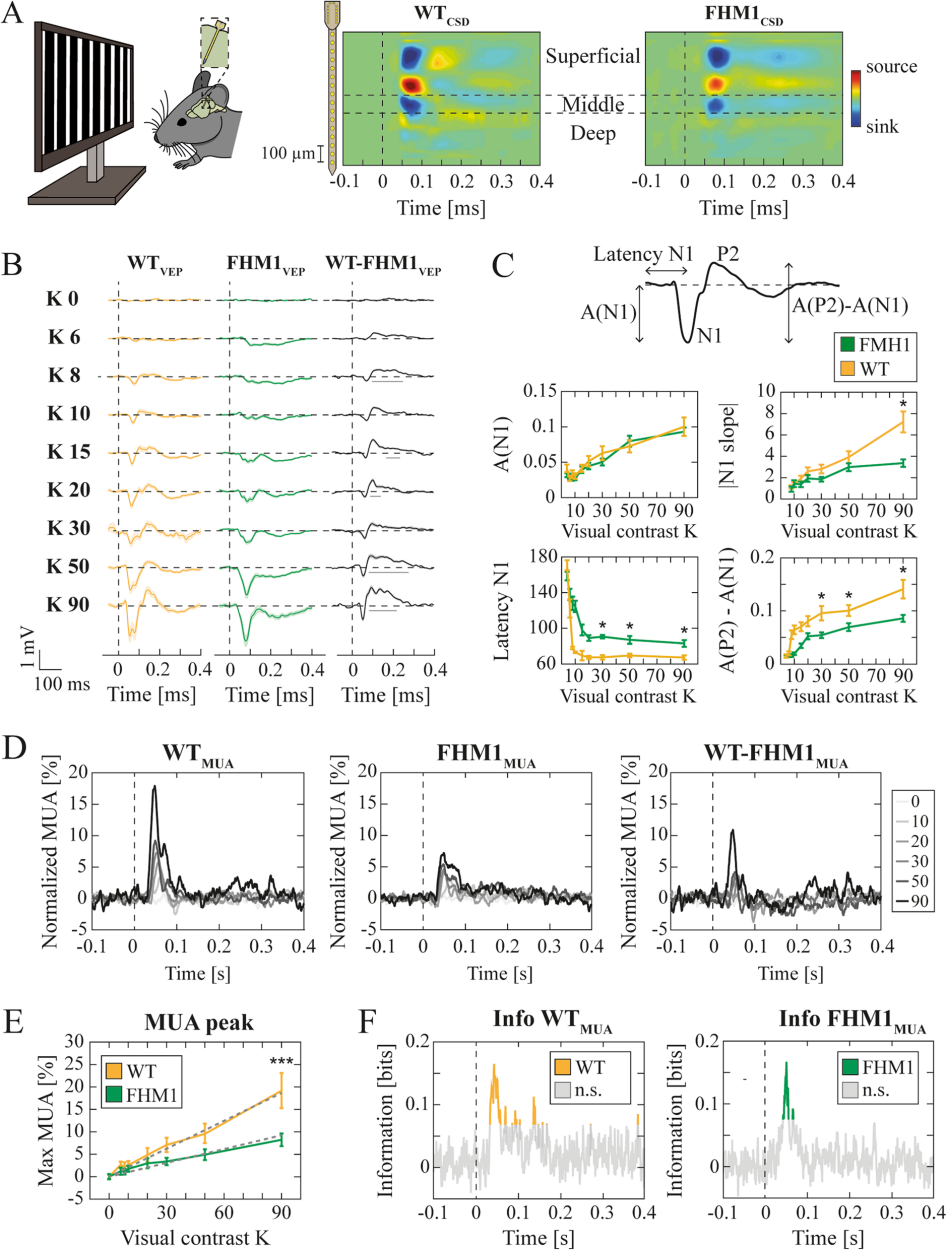
FHM1 mutations altered visual evoked potentials, decreased multi-unit activity but preserved information visual processing. **A** (left) Representative scheme of the experimental design. Square-wave 1 Hz alternating gratings at different contrast levels (K) were used for visual stimulation. A linear 16-channels probe (with 50 μm spacing between electrodes) was inserted into mice (*n* = 12 for WT; *n* = 12 for FHM1) V1. (right) Mean CSDs across animals aligned by the earliest current sink. **B** Mean VEPs across contrast levels K for WT (orange) and FHM1 (green) mice, and their difference (black). Solid horizontal lines indicate intervals of significant difference (permutation cluster-based test). Shaded regions indicate SEM. **C** (top) Schematic representation of the features extracted from the VEPs. (bottom) clockwise from top left to bottom right: amplitude of N1 [mV] (2WA: group F = 0.83; K F = 19.62; interaction F = 0.46); magnitude of the downslope of the first negative deflection of the VEPs [mV/s] (2WA: group F = 13.03; K F = 4.16; interaction F = 2.32); latency of N1 [ms] (2WA: group F = 175.78; K F = 28.56; interaction F = 4.63); amplitude difference between P2 and N1 [mV] (2WA: group F = 52.68; K F = 17.47; interaction F = 1.08). * *p* < 0.05 Dunn-Sidak post hoc test. **D** Normalized MUA of WT (left) and FHM1 (middle) mice and their difference (right) across contrast levels (in the legend). **E** Peak amplitude of normalized MUA of WT (orange) and FHM1 (green). 2WA K: F = 16.56; group: F = 12.98; interaction: F = 2.55. *** *p* < 0.001 Dunn-Sidak post hoc test. **F** Mutual information about contrast levels carried across time by the MUA of WT (left, orange) and FHM1 (right, green). Gray lines indicate non-significant (indicated as n.s. in the colorbar legend) MI (*p* > 0.05 bootstrap test)

Visual stimuli were generated according to the methods described in [18], and here summarized. Visual stimuli were computer-generated using the Matlab Psychophysics Toolbox with gamma correction and presented on a display (Sony; 40 9 30 cm; mean luminance 15 cd/m^2^) placed 25 cm from the head of the mouse (Fig. 1A), covering the center of the visual field. Extracellular signals were recorded in response to abrupt reversals (1 Hz) of vertical square-wave gratings (spatial frequency, 0.06 c/deg; contrast levels adopted ∈ [0,6,8,10,15,20,30,50,90]). Each visual stimulus at a given contrast was anticipated by a blank field lasting at least 3 seconds. Signals were amplified (5000-fold), bandpass filtered (0.5–500 Hz), and fed into a computer for storage and analysis. For each recording, alternating vertical gratings were presented for 30 seconds at a single contrast level to the head-fixed animals. To ensure consistency across animals and taking into account that there is little evidence for columnar organization of orientation-selective neurons in the mouse primary visual cortex [19], the orientation of the gratings was maintained vertical for all the recordings. Experiments were conducted blind regarding the genotype.

#### Recording implant

Animals were chronically implanted with a custom-made aluminum head post. A rectangular recording chamber (2 × 1.5 mm) of dental cement (Ivo-clar Vivadent Inc., USA) was built over the primary visual cortex at the following coordinates: between 0 and 1.5 mm anterior and between 1.5 and 3.5 lateral to the lambda suture. The skull was left intact. A ground electrode was placed over the cerebellum. The electrode was connected to a pin socket and secured to the skull by acrylic dental cement. Surgery was conducted under deep avertin anesthesia (7 ml/kg; 20% solution in saline, i.p.; Sigma Aldrich). Animals were then allowed to recover for 3 days. Following recovery, animals were progressively habituated for 3 days to the head fixation apparatus (Fisso, Zurich, Switzerland), as described [20]. A craniotomy overlying the primary visual cortex was performed under brief anesthesia by isoflurane 24 hours before the first recording session. To preserve the cortical surface, the recording chamber was filled with a layer of agar (Sigma Aldrich, USA) and the silicone elastomer Kwik (World Precision Instrument, USA.) as a protective cap. In order to discard non-visually evoked neural confounding, animals were restrained from moving while in the head fixation apparatus.

#### Extracellular recordings in awake mice

Recordings were performed on awake mice. Mice were carefully placed in the head fixation apparatus. After removing the protective cap, the recording chamber was filled with sterile saline solution (0.9%) in order to preserve and moisten the tissue*.*

A NeuroNexus Technologies 16-channel silicon probe (Fig. 1A) with a single-shank (A1x16-3 mm-50-177) was mounted on a three-axis motorized micromanipulator (Scientifica LTD, Uckfield, UK), placed in the central region of the recording chamber and slowly inserted into the visual cortex till the depth of 1000 μm. The electrode was then allowed to settle for about 5 min. The electrophysiological data were continuously recorded: LFPs were acquired at 1 kHz and bandpass filtered (0.3–200 Hz) using a 16- channel Omniplex recording system (Plexon, Dallas, TX, USA). At the end of the extracellular recording session, the recording chamber was covered with the protective cap as described above. Each animal underwent two recording sessions on two different days.

### Quantification and statistical data analysis

#### LFP and CSD analysis

We extracted the local field potentials (LFPs) by low pass filtering at 200 Hz the recorded extracellular signals. For each channel, visual evoked potential (VEP) waveforms in response to contrast reversals were extracted from the local field potentials (LFPs) by signal averaging (Fig. 1B, C). We extracted four features from the VEPs: (i) the amplitude of the negative peak (N1); the initial downslope measured as the slope from 2 to 25% of the N1 peak amplitude (as in [21]); the latency of N1; the difference in amplitudes between the later positive peak P2 and N1. For each recording session, the current source density (CSD, Fig. 1A) was computed by applying a standard algorithm considering the second spatial derivative estimate of the laminar LFP time series [22, 23] along with the iCSD toolbox for MATLAB [24]. A value of 0.3 S/m was taken as a measure of cortical conductivity (Gaussian Filter: standard deviation = 0.07 mm). Layer IV was identified in each recording session with the channel corresponding to the earliest current sink. The identified channels were independently confirmed to record from V1 layer IV (i) by visual inspection of the visual evoked potentials; (ii) by considering the penetration depth (in μm) provided by the micromanipulator at the end of electrode insertion.

#### Spectral analysis

LFPs were z-scored prior to spectral analysis. The power spectral density (PSD) of the z-scored LFPs was computed with the Fast Fourier Transform via the Welch method (pwelch function in Matlab), dividing the time window under investigation into sub-windows of 1000 ms with 50% overlap.

We expressed the spectral response of a given recording as the spectral modulation relative to the pre-stimulus response (consisting of a blank visual screen).

$${PSD}_{mod}\left(K,f\right)=\frac{PSD\left(K,f\right)-{PSD}_{pre- visual}\left(K,f\right)}{PSD_{pre- visual}\left(K,f\right)}$$

where K is the contrast level of the visual stimulus and f is the frequency.

**Fig. 2:**
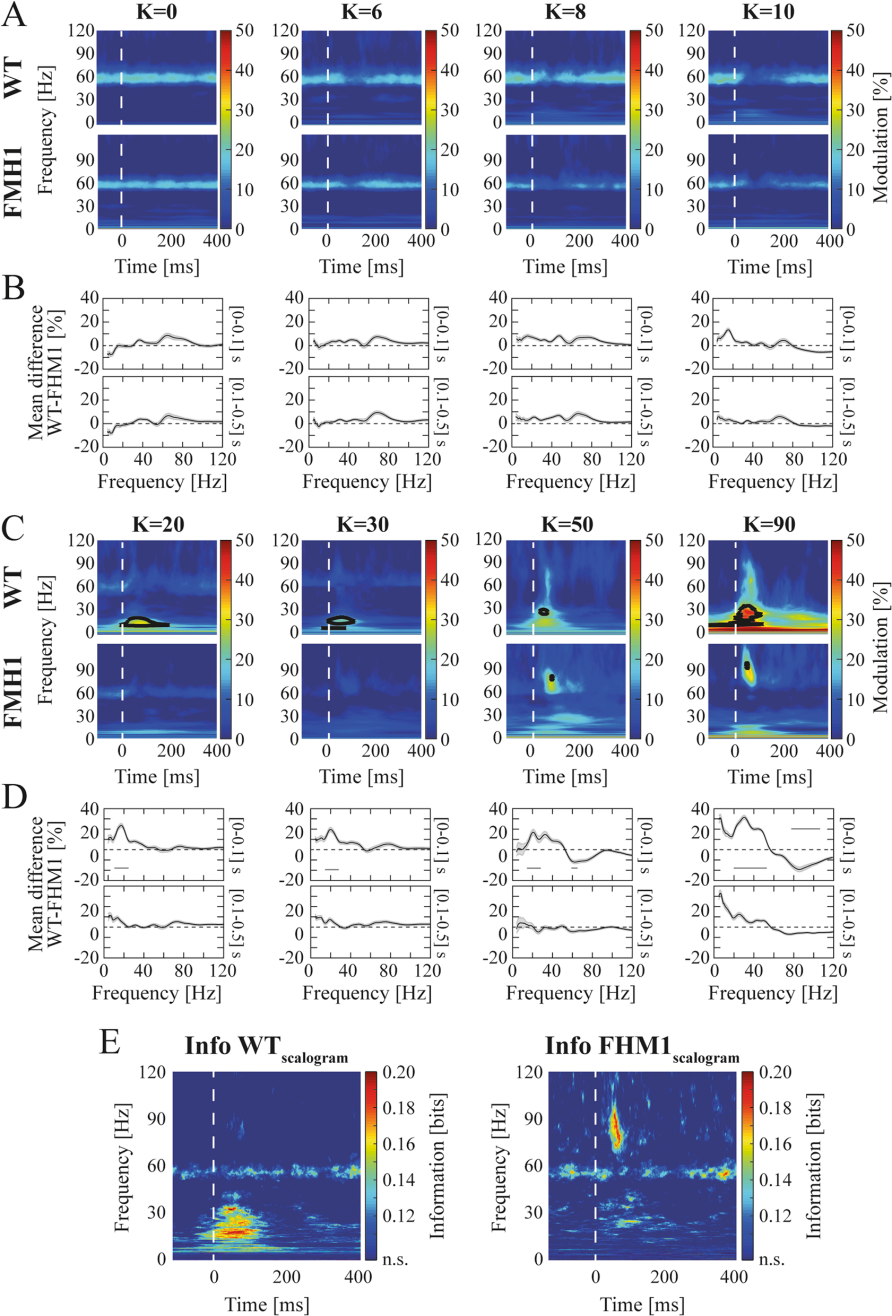
High visual contrasts were encoded in different ɣ bands in WT and FHM1 mice. **A** Mean scalogram modulation as a function of low visual contrasts (K ≤ 10) in WT (top row) and FHM1 (bottom row) mice around [− 100,400] ms of contrast reversals (indicated by the vertical dashed white lines for this whole figure). **B** Mean difference between the WT and FHM1 time-averaged scalogram modulations in the early ([0–100] ms, top row) and late ([200–500] ms, bottom row) window following contrast reversals. **C** Same as A) for high levels of visual contrast (K ≥ 20). Black contoured regions indicate statistical difference between WT and FHM1 (permutation pixel-based test). **D** Same as **B**) for high levels of visual contrasts (K ≥ 20). Statistical significance is depicted by solid horizontal lines (permutation cluster-based test). **E** MI carried by the LFPs scalogram modulations in WT (left) and FHM1 (right) mice about visual contrast levels. ‘n.s.’ in the colorbar stands for non-significant MI

We additionally computed the scalogram of the LFPs by means of wavelet analysis (*cwt* function in Matlab, Fig. 2). We used the analytic Morse wavelet with a symmetry parameter equal to 3 and the time-bandwidth product equal to 60. Scalograms were separately computed for each experimental recording and then split into 500 ms consecutive time windows (from − 100 to 400 ms around every square-wave contrast reversal).

As for the PSD modulation analysis, the LFPs scalograms were expressed as modulation with respect to the relative time-averaged pre-stimulus scalogram. Results of Fig. 2 were computed as the mean value of such scalograms across recordings and animals.

#### Multi-unit activity

Multi-unit activity (MUA) was extracted from the extracellular recordings by applying a bandpass filter (with stopband attenuation of 60 dB and with delay compensation introduced by the filter) and subsequently downsampled to 500 Hz. As for the LFPs spectral analysis, MUA was discretized into 500 ms consecutive time windows (from − 100 to 400 ms around every square-wave contrast reversal). To highlight the contrast-reversal-induced variation of firing activity, the MUA in each window was expressed as percentage variation with respect to the mean 100 ms MUA before the contrast reversal. The so computed MUA was called normalized MUA in the manuscript.

#### Mutual information

We computed the mutual information (MI, [25]) carried about visual contrast levels by (i) each time point describing the MUA evolution (Fig. 1F); (ii) each frequency-time tile composing the LFP scalograms (Fig. 2E). All information quantities were computed in Matlab with Information Breakdown Toolbox [26]. Probabilities estimates were computed by discretizing the observed responses into seven equi-populated bins. Limited dataset bias was accounted for by applying the Panzeri-Treves correction [27]. Significant information was estimated by applying a bootstrap procedure (with 500 iterations). The bootstrap procedure consisted of randomly pairing stimuli and responses. Alpha level was set to 0.05 and corrected with: (i) the Bonferroni method for multiple comparisons in the case of MUA evolution (Fig. 1F); (ii) the extreme pixel-based multiple comparison method [28] in the case of scalogram modulations (Fig. 2E).

#### Computational model

The simulated networks were adapted from previous works of our group [18, 29–32], for which we refer for more details. Briefly, the simulated networks consisted of *N* = 5000 leaky integrate and fire neurons [33]: 80% excitatory neurons (*N*_*E*_ = 4000) with AMPA-like synapses, and 20% inhibitory neurons (*N*_*I*_ = 1000) with GABA-like synapses [34]. The network is sparse and random, the connection probability between any directed pair of cells being 0.2 [35, 36]. The membrane potential *V*^*k*^ of each neuron k evolves according to [37]:

$${\tau}_m\frac{d{V}^k(t)}{dt}=-{V}^k(t)+{V}_{leak}+\frac{I_{tot}^k(t)}{g_{leak}}$$

where *τ*_*m*_ is the membrane time constant (20 ms for excitatory and 10 ms for inhibitory neurons), *g*_*leak*_ is the leak membrane conductance (25 nS for excitatory and 20 nS for inhibitory neurons), *V*_*leak*_ =  − 70 *mV*, and ${I}_{tot}^k(t)$ is the total synaptic input current. The latter was given by the sum of all the synaptic inputs entering the k-th neuron:

$${I}_{tot}^k(t)=\sum_{j\in AMPA}{C}_{jk}{I}_{AMPA}^k(t)+\sum_{j\in GABA}{C}_{jk}{I}_{GABA}^k(t)+{I}_{thal\_S}^k(t)+{I}_{thal\_ NB}^k(t)+{I}_{cort\ noise}^k(t)$$

Where *C*_*jk*_ ≠ 0 if neuron j projects to neuron k, and ${I}_{AMPA}^k(t)$, ${I}_{GABA}^k(t)$, ${I}_{thal}^k(t)$ and ${I}_{cort\ noise}^k(t)$ the different synaptic inputs entering the k-th neuron from recurrent excitatory, inhibitory, and thalamocortical synapses respectively.

The synaptic inputs currents were modeled as:

$${I}_{syn}^k(t)={g}_{syn}{s}_{syn}(t)\left({V}^k(t)-{V}_{syn}\right)$$

where *V*_*syn*_ are the synaptic reversal potential (*V*_*GABA*_ =  − 80 *mV* and *V*_*AMPA*_ = 0 *mV*) and *g*_*syn*_ are the synaptic conductances [38–41]. The conductances were set as follows (for the simulated WT mice): ${g}_{GABA^{inh}}^{WT}=2.70\ nS$ and ${g}_{GABA^{exc}}^{WT}=2.01\ nS$ for GABA-ergic inputs I_GABA_ to inhibitory and excitatory neurons respectively; ${g}_{AMPA^{inh}}^{WT}=0.233\ nS$ and ${g}_{AMPA^{exc}}^{WT}=0.178\ nS$ for recurrent AMPA-mediated inputs I_AMPA_ to inhibitory and excitatory neurons respectively; ${g}_{thal\_S\_{AMPA}^{inh}}^{WT}=0.317\ nS$ and ${g}_{thal\_S\_{AMPA}^{exc}}^{WT}=0.234\ nS$ for sustained thalamocortical AMPA-mediated inputs I_thal_S_ to inhibitory and excitatory neurons respectively; ${g}_{thal\_ NB\_{AMPA}^{inh}}^{WT}=0.317\ nS$ and ${g}_{thal\_ NB\_{AMPA}^{exc}}^{WT}=0.234\ nS$ for narrow band thalamocortical AMPA-mediated inputs I_thal_NB_ to inhibitory and excitatory neurons respectively; ${g}_{noise\_{AMPA}^{inh}}^{WT}=0.317\ nS$ and ${g}_{noise\_{AMPA}^{exc}}^{WT}=0.234\ nS$ for cortical noise AMPA-mediated inputs I_cort_noise_ to inhibitory and excitatory neurons respectively.

We summarized the FHM1 synaptic alteration observed experimentally [15, 16] into three factors: increase of (i) intra-cortical (IC) and (ii) thalamocortical (TC) AMPA-mediated synapses; (iii) the thalamocortical synaptic increase is higher in inhibitory rather than excitatory neurons (in the main text this phenomenon was called thalamocortical synaptic asymmetry, TCA). We implemented such alterations in our computational model by increasing the aforementioned conductance levels of those synapses involved in the FHM1 cellular alteration. Specifically:

(i) IC increase was simulated by:

$${g}_{AMPA^{inh}}^{FHM1}={g}_{AMPA^{inh}}^{WT}\left(1+\frac{C{C}_{incr}\%}{100}\right)\ \mathrm{and}\ {g}_{AMPA^{exc}}^{WT}={g}_{AMPA^{exc}}^{FHM1}\left(1+\frac{C{C}_{incr}\%}{100}\right)\frac{}{}$$

(ii and iii) TC increase and TCA were simulated by:

$\alpha =1+\frac{TCA\%}{100}$; ${g}_{thal\_S\_{AMPA}^{inh}}^{FHM1}=\left(1+\alpha \frac{2\frac{T{C}_{incr}\%}{100}}{1+\alpha}\right){g}_{thal\_S\_{AMPA}^{inh}}^{WT}$ and ${g}_{thal\_S\_{AMPA}^{exc}}^{FHM1}=\left(1+\frac{2\frac{T{C}_{incr}\%}{100}}{1+\alpha}\right){g}_{thal\_S\_{AMPA}^{exc}}^{WT}$. *CC*_*incr*_ % , *TC*_*incr*_ % and *TCA*% are the factors, expressed as percentage, representing the corresponding FHM1 cellular alterations.

The time course of synaptic currents, i.e., *s*_*syn*_(*t*), was incremented by an amount described by a delayed difference of exponentials every time a pre-synaptic spike occurred at time *t*^∗^ [37]:

$$\Delta {s}_{syn}(t)=\frac{\tau_m}{\tau_d-{\tau}_r}\left[\exp \left(-\frac{t-{\tau}_l-{t}^{\ast }}{\tau_d}\right)-\exp \left(-\frac{t-{\tau}_l-{t}^{\ast }}{\tau_r}\right)\right]$$

where the latency *τ*_*l*_ represented the axonal delay, and *τ*_*r*_ and *τ*_*d*_ represented respectively the rise and decay time of the post-synaptic currents. The time constants values were set as follows [38, 40, 42–45]: the latency *τ*_*l*_ was set to 1 ms and 2 ms for GABA-ergic and AMPA-like synapses respectively; the rise time *τ*_*r*_ was set to 1 ms, 0.2 ms and 0.4 ms for GABA-ergic, AMPA-like on excitatory, and AMPA-like on inhibitory synapses respectively; the decay time *τ*_*d*_ was set to 5 ms, 1.25 ms and 2.25 ms for GABA-ergic, AMPA-like on excitatory and AMPA-like on inhibitory synapses respectively.

All neurons received three external (meaning non-recurrent) inputs ${I}_{thal\_S}^k(t)+{I}_{thal\_ NB}^k(t)+{I}_{cort\ noise}^k(t)$. ${I}_{thal\_S}^k(t)$ is an excitatory input representing the sustained component of thalamocortical afferents [18] that increases with visual contrast. ${I}_{thal\_S}^k(t)$ was implemented as a series of spike times that activated excitatory synapses with the same kinetics as recurrent AMPA synapses, but different strengths (see *g*_*syn*_ values reported above). These synapses were activated by independent realizations of random Poisson spike trains, with a rate *v*_*ext* _ *thal* _ *S*_ = [*S*(*K*)]_+_ identical for all neurons.

${I}_{thal\_ NB}^k(t)$ is another excitatory input that simulated the narrow ɣ band component of thalamocortical afferents [18, 46] that decreases with visual contrast. The Poisson spike trains that simulate ${I}_{thal\_ NB}^k(t)$ had a rate *v*_*ext* _ *thal* _ *NB*_ = [*A*(*K*, *t*)*ε*_*γ*_(*t*)]_+_. *A*(*K*, *t*) is the amplitude of a ɣ range filtered white constant noise *ε*_*γ*_(*t*). *ε*_*γ*_(*t*) was obtained by applying a 3rd-order bandpass Butterworth filter of central frequency equal to 57 Hz and bandwidth equal to 10 Hz to white noise [18].

*A*(*K*) and *S*(*K*) values were chosen as to maximize the agreement with WT experimental data [18]. Specifically: *A*(*K* ≥ 30) = 0 *sp*. /*s* and *S*(*K* ≤ 30) = 1000 *sp*. /*s*; *A*(*K* = 0, 6, 8,10,20) = [50,45,40,30,15] *sp*. /*s* and *S*(*K* = 30,50,90) = [1000,1040,1080] *sp*. /*s*; *S*(*pre* − *visual stim*.) = 1000 *sp*. /*s* and *A*(*pre* − *visual stim*.) = 0 *sp*. /*s*.

${I}_{cort\ noise}^k(t)$ is colored noise mimicking stimulus-unspecific cortical activity. Its spike times were simulated with a Poisson process with rate *v*_*cort* _ *noise*_ = [*ϑ*_*n*_*n*(*t*)]_+_. The noise term *n*(*t*) is a z-scored colored noise, with the PSD following *S*(*f*) = 1/*f*^1.5^, and an amplitude factor *ϑ*_*n*_ = 0.4 *sp*. /*ms*. […]_+_ is a threshold-linear function, [𝑥] + =𝑥 if 𝑥> 0, [𝑥] + =0 otherwise.

The LFPs of the simulated networks were estimated as the sum of the absolute value of the GABA and AMPA currents (both external and recurrent) that enter all excitatory neurons [47].

Network simulations were performed using a finite difference integration scheme based on the second-order Runge Kutta algorithm [48, 49] with time step Δ𝑡=0.05 𝑚𝑠. To focus on stationary responses, the first 200 ms of every simulation were discarded. Simulations were set to simulate 5 seconds of neuronal activity. Each network parameter combination was simulated 40 times.

All simulations were conducted with custom-made Python scripts within the Brian 2 simulator environment [50, 51].

#### Inhibitory over excitatory ratio

The recurrent inhibitory over excitatory ratio (*IEratio*_*rec*_ of Fig. S1 I-M) of the simulated neuronal network was estimated as:

$${IEratio}_{rec}=\frac{1}{N_E}\sum_{k\in Exc}\left\{\sum_t\frac{\sum_{j\in AMPA}{C}_{jk}{I}_{AMPA}^k(t)}{\sum_{j\in GABA}{C}_{jk}{I}_{GABA}^k(t)}\right\}$$

**Fig. 3:**
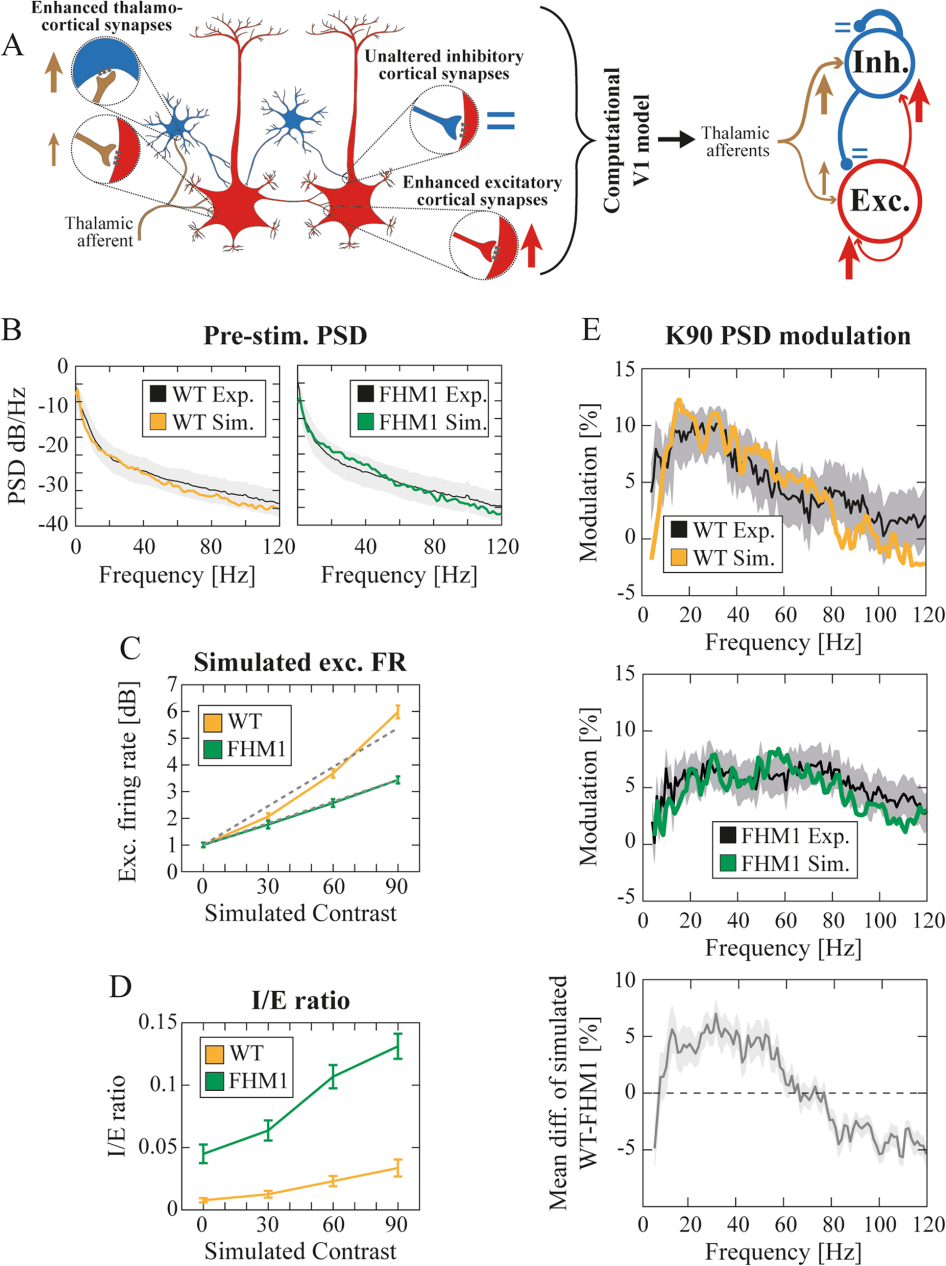
Spiking neuron network embedding the FHM1 synaptic alterations. **A** (left) Schematic representation of the synaptic alterations experimentally observed in [15, 16] (left) and implemented in our computational model (right). **B** Mean PSD of experimental and simulated LFPs for WT (left) and FHM1 (right) mice during pre-stimulus baseline. The shaded regions indicate the standard deviation of experimental PSDs. **C** Simulated normalized MUA of WT (orange) and FHM1 (green) model. **D** Inhibitory over excitatory ratio in the simulated WT (orange) and FHM1 (green) computational model across simulated K. 2WRMA, K: F > 1000; group: F > 1000; interaction: F = 646.2. **E** (top and middle) PSD modulation for the maximum contrast level (K = 90) of experimental (black) and simulated data for WT (orange, top) and FHM1 (green, middle). See also Fig. S1 A-B for responses to low contrasts. (bottom) Mean difference between the simulated WT and FHM1 spectral modulations (shaded error bar indicate standard deviation of bootstrapped mean difference)

**Fig. 4:**
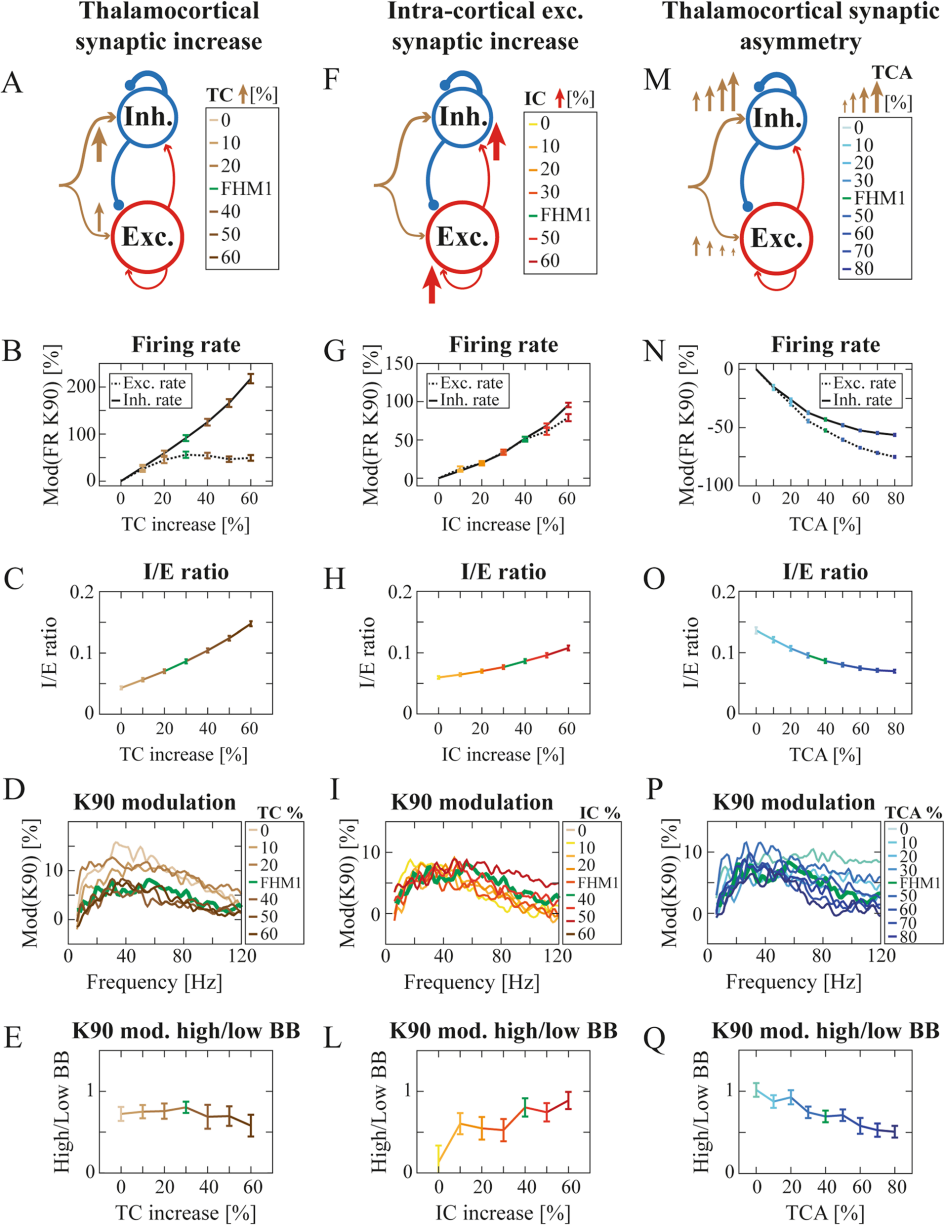
Cortico-cortical and thalamocortical gain-of-function and thalamocortical asymmetry differentially contributed to shaping cortical dynamics in FHM1 computational model. **A** Schematics of the FHM1 computational model when considering only the thalamocortical (TC) synaptic increase (represented by sand-colored arrows with thickness proportional to synaptic strength). **B** Modulation of excitatory (dashed black line) and inhibitory (black line) firing rate across TC synaptic increase levels (mean ± std). The modulation was computed with respect to the simulated pre-stimulation baseline firing rate. 2WRMA TC: F = 474.85; neuronal type: F = 25,579; interaction: F = 3291.9. See also Fig. S1 C. **C** Inhibitory over excitatory ratio (averaged across contrast levels) in the simulated excitatory neurons across TC increase levels (mean ± std). 2WRMA TC: F > 1000; K: F > 1000; interaction: F = 49.30. See also Figs. S1 F, I, S3 E. **D** PSD modulations of the simulated LFPs at K = 90 with respect to pre-stimulation across TC increase levels. **E** Ratio of high over low broad ɣ band of PSD modulation of simulated LFPs across TC increase levels. See also Fig. S3 B-D, F. **F**-**L** Same as (**A**-**E**) but across IC increase levels. **G** 2WRMA IC: F = 293.93; neuronal type: F > 1000; interaction: F = 614.09. See also Fig. S1 D. **H** 2WRMA K: F > 1000; IC: F = 585.83; interaction: F = 44.53. See also Fig. S1 G,L, S3 M. **L** See also Fig. S3 H-L, N. **M**-**Q** Same as (A-E) but across TCA levels. **N** 2WRMA TCA: F = 649.57; neuronal type: F > 1000; interaction: F = 251.39. See also Fig. S1 E. **O** 2WRMA TCA: F > 1000; K: F > 1000; interaction: F > 1000. See also Figs. S1 H, M, S3 S. **Q** See also Fig. S3 P-R, T

The inhibitory over excitatory ratio (*IEratio* of Figs. 3D, 4CHO, S1 F-H), including also non-recurrent synaptic sources, was estimated as:

$$IEratio=\frac{1}{N_E}\sum_{k\in Exc}\left\{\sum_t\frac{\sum_{j\in AMPA}{C}_{jk}{I}_{AMPA}^k(t)+{I}_{thal\_S}^k\left({t}_i\right)+{I}_{thal\_ NB}^k(t)}{\sum_{j\in GABA}{C}_{jk}{I}_{GABA}^k(t)}\right\}$$

#### Permutation statistical analysis

We decided to adopt non-parametric permutation statistical analysis throughout the manuscript to avoid, when possible, any apriori assumptions on the data. Furthermore, appropriate corrections for multiple comparisons can easily be incorporated into such analysis [52].

Throughout the manuscript, we applied two different multiple comparison correction strategies [28]: (i) pixel-based for two-dimensional data (in our case, time-frequency points of scalogram modulations of Fig. 2A, C); (ii) cluster-based for one-dimensional data (in our case, VEPs of Fig. 1B, temporal average of scalogram modulations of Fig. 2B, D, and power spectral density of pre visual activity of Fig. 3B). In both cases, scalogram modulations were pooled across animals and recordings.

Correcting for multiple comparisons using pixel-based statistics involves creating a null distribution containing the most and least pixel extreme values (i.e., the minimum and maximum value of time-frequency tile of scalogram modulations). The 2.5th and 97.5th percentile of this null distribution were consequently chosen as corrected alpha values of a two-tailed test.

As with pixel-based correction, cluster-based statistics involve generating a null distribution. At each iteration of permutation testing, a threshold is applied to the permuted data at an uncorrected alpha level of 0.05. The maximal length of consecutive significant values for each iteration is then retained and considered as a proxy for the multiple comparison null distribution. Hence, only those clusters of significant values in real data that were larger(lower) than 97.5th (2.5th) percentile of this null distribution.

## Results

### FHM1 mutation decreased multi-unit activity but not its information content

To investigate the alterations in V1 dynamics induced by the FHM1 mutation, we compared the neural activity recorded in awake head-fixed WT (*n* = 12) and R192Q FHM1 knock-in mice (*n* = 12) [12] (see Methods) during the presentation of the abrupt reversal of visual gratings spanning a broad range of spatial contrast levels K (Fig. 1A left, see Methods for details). The activity was recorded from multiple depths with a 16 channels linear probe but here we will focus on the recordings acquired from layer IV (Fig. 1A middle and right, see Methods), which is the recipient of most thalamocortical projections [53].

Visual evoked potentials (VEPs) in FHM1 mice displayed a significantly different temporal evolution from WT (Fig. 1B, right column, *p* < 0.05 after ~ 100 ms from contrast reversal for K ∈ [8 15 20 50 90], permutation cluster-based test, see Methods). Decomposing VEPs into stereotypical reference points [54–56] (Fig. 1C, top) showed that i) the amplitude of the negative VEPs peak (N1) was similar in the two animal groups (Fig. 1C top-left. Two-way ANOVA (2WA) group: *p* = 0.36; K: *p* < 0.001; interaction: *p* = 0.87); ii) N1 downslope was less steep in FHM1 (Fig. 1C top-right. 2WA group: *p* < 0.001; K: *p* < 0.001; interaction: *p* = 0.03), iii) N1 peak latency was significantly larger in FHM1 (Fig. 1C bottom-left. 2WA group: *p* < 0.001; K: *p* < 0.001; interaction *p* < 0.001), iv) the later positive peak (P2, occurring at ~ 150 ms after visual contrast reversal) was observed only in WT mice (Fig. 1C bottom-right. 2WA group: *p* < 0.001; K: *p* < 0.001; interaction: *p* = 0.37). The lack of P2 in FHM1 VEPs also unmasked a later negative VEP component.

The peak of the Multi-Unit Activity (MUA, see Methods) occurred at 64.03 ± 4.92 ms for WT and 69.84 ± 4.29 ms for FHM1 mice after contrast reversal (mean ± standard error of the mean, SEM) and was less pronounced in FHM1 mice at high contrasts (Fig. 1D). The MUA peak amplitude grew linearly with K for both groups, with a steeper slope for WT (Fig. 1E. 2WA K: *p* = 0.001; group: *p* = 0.002; interaction: *p* = 0.01): Slope WT = 0.2 ± 0.04 (mean ± std. bootstrap), *p* < 0.001 t-test for linear regression slope; slope FHM1 = 0.09 ± 0.01, *p* < 0.01; slope ratio = 2.3 ± 0.6 (mean ± 95% confidence interval). The reduced MUA peak was coherent with an I/E ratio shifted toward inhibition. Despite such reduced modulation range, the MUA carried the same amount of mutual information (MI, see Methods, [57]) about visual contrasts in the two groups (Fig. 1F, MI maxima: 0.17 bits at 44 ms for WT and 0.17 bits at 50 ms for FHM1).

### FHM1 mutation shifted contrast encoding towards a faster gamma range

We next investigated whether FHM1 mutations altered the spectral proprieties of V1 local field potentials (LFPs) responses to visual contrasts. At baseline (i.e., pre-stimulation consisting of a blank visual stimulus) the spectra were similar between the two groups (*p* > 0.05 permutation cluster-based test). For contrast levels up to K ≤ 10, both groups showed a prominent component in the narrow ɣ band range (Fig. 2A, 56.7 ± 0.6 Hz, mean ± SEM, *p* > 0.05, permutation pixel-based test) whose power decreased with visual contrast, as in [18, 46]. In this contrast range, the two groups showed no significant difference over the whole spectrum (Fig. 2B, *p* > 0.05 for K ≤ 10, both early and late response phase, permutation cluster-based test). For K ≥ 20, the spectral responses of the two groups increasingly diverged along with the contrast level. WT mice displayed an increase in the β/low ɣ component ([12-40] Hz, Fig. 2C top row) as in [18]. Instead, FHM1 mice displayed a high ɣ band increase ([70–100] Hz, Fig. 2C bottom row). As both spectral modulations occurred mostly in the first 100 ms after contrast reversal, the overall spectrum was significantly different between the two groups within this temporal interval (Fig. 2C, black contoured regions indicate statistical differences with permutation pixel-based test. Figure 2D top row, *p* < 0.05 for all contrasts in the early response, permutation cluster-based test). The frequency range encoding information about high contrasts transitioned from the β/low ɣ range in WT (Fig. 2C top row and E left) to the high ɣ range in FHM1 mice (Fig. 2C bottom row and 2E right), in both cases within the early response phase. The amount of information carried by LFP modulation in the two groups was similar (0.14 bits in the β/low ɣ range in WT and 0.10 bits in the high ɣ band in FHM1). Information about low contrasts carried by the narrow band (Fig. 2A) was also similar (0.11 bits in WT and 0.12 bits FHM1). These results corroborate the hypothesis of altered, but information conservative, dynamics of contrast encoding in the FHM1 visual cortex.

### A computational model linking FHM1-induced synaptic modifications with altered visual cortex contrast encoding

We next investigated the link between the results displayed above and the FHM1 synaptic alterations [10, 15–17] to understand how the observed pathological responses are compatible with an increase in both thalamocortical and intra-cortical glutamatergic synaptic strength [15, 16].

To model V1 in FHM1 we adapted a computational model of WT mice V1 [18] (see Methods) by introducing the FHM1-induced synaptic changes as follows: i) intra-cortical and thalamocortical glutamatergic transmission strength were enhanced (as experimentally observed in microcultures and brain slices in [15–17]); ii) the thalamocortical gain-of-function was set to be higher on cortical GABAergic as compared to excitatory neurons (as experimentally observed in slices in [16]) (Fig. 3A, see Discussion and Methods).

Please note that for the lack of thalamic recordings and the sake of simplicity, the thalamocortical input to the simulated network was assumed to be time-invariant and with the same firing rate in WT and FHM1 mice (see Methods and Limitations of the study in the Discussion). The simulated results described onwards are therefore to be interpreted as the temporal average (i.e., the average across the whole contrast reversal interval) of the experimental results presented in the previous sections.

The FHM1 V1 model reproduced (i) the pre-stimulus baseline PSD LFPs (Fig. 3B); (ii) the experimentally observed ratio between the firing rate in WT and FHM1 over the inspected contrast levels (Fig. 3C. Slope ratio 1.91 ± 0.40 (mean ± 95% bootstrap confidence interval), compare with Fig. 1E). The simulated I/E ratio (see Methods) increased with contrast, as in [58], in both groups but was shifted towards inhibition in FHM1 mice (Fig. 3D, Two-way repeated measure ANOVA (2WRMA), K: *p* < 0.001; group: *p* < 0.001; interaction: *p* < 0.001. See also Fig. S 3).

The model also reproduced the similarity of the LFPs spectral modulation (i.e., the spectral content of the LFPs over the contrast reversal interval with respect to baseline, see Methods) in the two groups for low contrasts (Fig. S 1A). In addition, the model quantitatively reproduced both WT and FHM1 spectral modulation at high contrasts (Fig. 3E top and middle, for K = 90, ${\chi}_r^2=0.25$ for WT and ${\chi}_r^2=0.23$ for FHM1). In particular, in our simulations, in FHM1 mice the spectral modulation was weaker than in WT in the low gamma band and stronger in the high gamma band (Fig. 3E, bottom), coherently with what was observed in experimental data (Fig. 2D, last column on the right, top panel). Note that such changes in spectral modulation in FHM1 mice might be due to a small extent to a slightly higher baseline (Fig. 3B right), but mostly to a change in spectral power of the simulated LFP at K = 90 (Fig. 3E). Overall, our simple model of V1 could capture a wealth of migraine-driven network effects, once FHM1 synaptic modifications were properly implemented.

### Complementary effects of thalamocortical and intra-cortical FHM1 synaptic modifications on visual cortex dynamics

As described above we modeled FHM1 dynamics as a result of three synaptic variables: i) thalamocortical transmission (TC), ii) intra-cortical excitatory transmission (IC), iii) thalamocortical transmission asymmetry (TCA). We then investigated the relative contribution of each of these variables separately, while keeping the other two at the values leading to the optimal agreement with experimental data shown in Fig. 3.

Both excitatory and inhibitory firing rates increased with TC modulation, but with a significantly stronger effect on inhibitory firing rate (Fig. 4B, 2WRMA TC: *p* < 0.001; neuron type: *p* < 0.001; interaction: *p* < 0.001. The frequency rate increase at reference TC = 30% was equal to 90.9% and 53.3% for inhibitory and excitatory neurons respectively. See also Fig. S1C). TC also increased the I/E ratio (Fig. 4C. 2WRMA TC: *p* < 0.001; K: *p* < 0.001; interaction: *p* < 0.001. See also Figs. S1F, I, and S3E). TC affected the overall magnitude but not the shape of the LFP spectral modulation (Fig. 4D) and hence did not significantly affect the ratio between high and low ɣ broad band (BB) (Fig. 4E. Friedman test *p* = 0.79. See also Fig. S 3F).

Modulation of IC (Fig. 4F) also significantly increased both inhibitory and excitatory firing rates but with a milder effect on their difference (Fig. 4G. 2WRMA IC: *p* < 0.001; neuronal type: *p* < 0.001; interaction: *p* < 0.001. The frequency rate increase at reference IC = 40% was equal to 51.1% and 49.8% for inhibitory and excitatory neurons respectively. Note however the absolute increase is larger for inhibitory neurons, Fig. S1D). IC increase led to a significant increase in the I/E ratio (Fig. 4H. 2WRMA K: *p* < 0.001; IC: *p* < 0.001; interaction: *p* < 0.001. See also Fig. S1G. See Figs. S1L and S3M for the recurrent I/E ratio) associated to increase of the inhibitory firing rate. IC modulation affected LFP spectral modulation (Fig. 4I), significantly increasing the ratio between high and low ɣ BB (Fig. 4L. Friedman test *p* < 0.001. See also Fig. S3N).

These results suggest that TC enhancement is responsible for driving the network towards a more inhibited state, by preferentially increasing the firing rate of inhibitory neurons due to TCA (Figs. 4B, S1C). TC increase altered the overall LFP spectral power but not the relative contribution of the high and low ɣ range (Fig. 4E). IC enhancement, instead, increased similarly the firing rate of both neuronal types (Figs. 4G, S2C), and induced a shift toward a higher ɣ range (Fig. 4L). Note that removing thalamocortical increase asymmetry (TCA = 0) led to a dramatic decrease in the robustness of the agreement with experimental data to variations in TC and IC (Fig. S2). An increase in TCA (Fig. 4M) led to an decrease in both excitatory and inhibitory firing rates (Fig. 4N. 2WRMA TCA: *p* < 0.001; neuronal type: *p* < 0.001; interaction: *p* < 0.001. The frequency rate decrease at reference TCA = 40% was equal to − 43.7% and − 52.8% for inhibitory and excitatory neurons respectively. See also Fig. S1E). The I/E ratio decreased with increasing TCA (Fig. 4O. 2WRMA TCA: *p* < 0.001; K: *p* < 0.001; interaction: *p* < 0.001. See also Fig. S1H for the recurrent I/E ratio) due to decreased inhibitory activity. TCA also affected the shape of the LFP spectrum (Fig. 4P): the more symmetrical the thalamocortical transmission the more similar the modulation exerted by visual contrast on high and low BB power (Fig. 4Q. Friedman test *p* < 0.001. See also Fig. S3T).

## Discussion

We characterized alterations of V1 dynamics in FHM1 mice with novel experimental observations. At contrast reversals, VEPs were slower, and MUA was less intense compared to WT mice (Fig. 1). LFPS spectral modulation in response to high contrasts showed a shift from low to high ɣ frequency compared to WT mice (Fig. 2). To understand the underpinnings of these novel experimental results, we embedded the synaptic alterations found in the FHM1 genetic mouse model [15–17] in a V1 spiking network model previously found to reproduce ɣ dynamics in rodents [18]. The model was able to replicate the aforementioned experimental results if and only if both IC and differential (i.e., larger on inhibitory neurons) TC synaptic alterations were included (Fig. 3). In particular, the model suggested that the I/E ratio increased even in the presence of stronger IC glutamatergic synapses because this was overridden by the larger gain-of-function of TC synapses onto inhibitory compared to excitatory neurons. The frequency shift was instead due to the increase in IC glutamatergic transmission (Fig. 4L). Understanding the specific role of each synaptic modification could help disentangle cortical and thalamic roles in migraine symptoms [59, 60] and might be critical in the development of future drugs.

The observed decrease in MUA response of FHM1 mice (Fig. 1D-E) suggests an increase in the I/E ratio, coherently with our simulations (Fig. 3C-D). However, the amount of information about contrast carried by firing activity in FHM1 V1 was the same as in WT (Fig. 1F), even if the modulation range was narrower. Also, LFPs spectral information (Fig. 2E) was not quantitatively altered in FHM1, even if the information peak in FHM1 moved towards higher frequencies. Interestingly, modulation of ɣ oscillations at the high-end of the band can actually be even more efficient to decode post-synaptically, as inputs have more impact on a postsynaptic target neuron if they coincide within an interval comparable with the neurons’ integration window [61–64]. Hence the shift towards higher frequencies that we observed may be compatible with the enhanced sensitivity to visual stimulation observed in migraineurs [65, 66].

While our findings might be relevant to explain some of the alterations in sensory processing found in FHM patients, it seems difficult to envision how they might be relevant to explain their increased susceptibility to ignition of cortical spreading depression, the phenomenon underlying migraine aura [67]. Threshold levels of glutamate, glutamatergic plumes and activation of glutamate NMDA receptors in the apical dendrites of cortical pyramidal cells appear necessary for ignition of experimental cortical spreading depression, which is facilitated in FHM mouse models because these threshold levels are reached at a lower stimulation intensity as a consequence of increased IC glutamatergic neurotransmission [15, 68, 69]. These findings suggest that ignition of a “spontaneous” cortical spreading depression in the FHM brain could be favoured by conditions leading to excessive synaptic excitation of distal dendrites resulting in their depolarization and local elevation of glutamate above the threshold level necessary for activation of a sufficient number of dendritic NMDA receptors. Much work remains to be done in the FHM mouse models to uncover the conditions in which this may occur in response to specific migraine triggers.

FHM1 and WT mice displayed similar spectral responses to low contrasts stimuli (Figs. 2B, S1A). This can be explained by combining two observations. (i) in mice V1, LFPs are dominated by a ɣ narrow band (peaking at ~ 60 Hz) at low contrasts. This narrow band reflects postsynaptic TC currents on excitatory neurons entrained by the ~ 60 Hz rhythmic firing of lateral geniculate nucleus neurons [18, 46] (ii) In FHM1 mice postsynaptic TC currents are enhanced. However, the fact that short-term depression of thalamocortical synapses on excitatory neurons is greater in FHM1 as compared to WT mice [16], implies that the gain-of-function of TC currents on excitatory neurons is greatly reduced for the steady 60 Hz thalamic input responsible for the cortical ɣ narrow band. As shown in Fig. S1B, removing this assumption would lead to simulated results not matching experimental observations.

The differences in visual contrast encoding between the two groups unfold only for high contrasts and within the early (~ 100 ms) response window. High contrasts are known to increase global ɣ synchronization in the visual cortex [70]. For high levels of synchronization the network displays endogenous oscillations depending both on the external input and its resonant proprieties [30, 37, 71]. These oscillations frequencies are known to be dependent also on IC synaptic strengths, instead not involved in the generation of cortical ɣ narrow band for low contrasts. Consequently, FHM1 synaptic alterations redesign the pathological network more prominently at high contrasts, sculpturing its activity to become divergent from the physiological WT scenario.

Although differences in stimulation design and recordings procedure prevent direct comparison with our findings, it is interesting that a recent study of the EEG responses to flash light stimulation at different frequencies revealed enhancement of photic driving for stimulation frequencies in the beta-gamma band in freely behaving FHM1 mice [72].

### Limitations of the study

This work aimed to investigate the change in visual cortex circuitry gamma-band modulation caused by the synaptic alterations characterizing FHM1 by probing them with visual stimulations composed of grating bars of different levels of contrast. Nonetheless, visual contrast is far from being the only stimulus feature modulating cortical activity and gamma oscillation. Visual gamma oscillations have indeed been shown to be modulated by luminance, color, orientation, temporal frequency, stimulus size, and so on ( [73] and references therein). Further studies should therefore investigate to what extent the FHM1 alterations of visual processing presented here still holds and/or are modified when modulating different visual stimuli features. Moreover, in our work, we did not take into account behavioral factors (e.g., locomotion) known to influence gamma oscillation in response to visual stimulation [46, 74].

We focused on gamma oscillations as they are widely accepted to be one of the strongest markers of visual information processing in the visual cortex, also reflecting the modulation in single units firing activity [75] that were too sparse to be properly isolated in our recordings.

As far as it concerns our computational model, one of the main limitations is that we addressed only averaged proprieties of the neural responses. The rationale behind this computational design choice can be found in our lack of experimental data about the instantaneous dynamics of thalamic afferents in the visual cortex. For the sake of simplicity, we assumed then the thalamic afferents to be represented by a steady, time-invariant, firing rate throughout visual stimulation. The simulated results are therefore to be interpreted as the temporal average (i.e., the average across the whole contrast reversal interval) of the experimental results presented in Figs. 1 and 2. The lack of temporal dynamics in the thalamocortical afferents explains, furthermore, the reasons why our model is not suited to replicate the VEPs differences between the two groups. It is not possible, indeed, to simulate visual evoked potentials following contrast reversals without knowing the temporal dynamics of thalamic inputs into V1. Such a lack of experimental data also justifies the assumption of thalamocortical afferent activity to be equal between WT and FHM1 mice. Future studies will address these limitations to disentangle the contribution to the visual processing abnormalities here reported between subcortical and cortical mechanisms in FHM1 mice.

Further model extensions could be: (i) the addition of different interneuron types (such as somatostatin- or vasointestinal peptide-expressing) that might play a role in FHM1 [76–78]; (ii) neuronal connectivity dependent both on the inter-somatic distance and on the neuronal type [79, 80].

## Conclusions

By combining experimental and simulated data analysis, our work identified candidate mechanisms linking FHM1 synaptic alterations with network-wide pathological cortical dynamics in V1. Our approach to investigating the link between these two spatial scales (micro- and meso-scopic) could also be extended beyond the specific pathological state here addressed. Furthermore, the computational model could also offer a promising benchmark for developing novel pharmacological targets and predicting in silico their effects in the network processing of sensory information.

## Supplementary Information

Additional file 1:**Fig. S1 related to Figs. 3, 4.** (A) (left) PSD modulation for the minimum contrast level (K = 0) of experimental (black) and simulated data for WT (orange, left) and FHM1 (green, right). ${\chi}_r^2=0.34$ for WT and ${\chi}_r^2=0.28$ for FHM1. (B) Cortical narrow band power modulation as a function of thalamic narrow band strength in the FHM1 model. The model was set with (red) and without (dark blue) waning of FHM1 thalamocortical strengthening at the high rate of LGN neurons generating NB (mean ± SEM). This phenomenon was simulated by injecting the thalamic NB in thalamocortical synapses with (blue) or without (red) the gain-of-function induced by the FHM1 mutations. 2WRMA: Thalamic NB: *p* < 0.001, F = 1666; presence/absence of TC increase: *p* < 0.001, F = 157.45; interaction: *p* < 0.001, F = 43.28. (C) Excitatory (dashed black line) and inhibitory (black line) simulated firing rate across TC increase levels (mean ± std). (D) Same as C) but across IC synaptic increase levels. (E) Same as C) but across TCA. (F) Inhibitory over excitatory ratio (mean ± std) in the simulated excitatory neurons across TC increase levels (in the legend). Please note the difference with respect to the recurrent I/E ratio of Fig. 3D, 4CHO (See Methods). (G) Same as F) but across IC increase levels (indicated in the legend). (H) Same as F) but across TCA levels (indicated in the legend). (I) Recurrent inhibitory over excitatory ratio (mean ± std) in the simulated excitatory neurons across TC increase levels (in the legend of panel F). (L) Same as I) but across IC increase levels. (M) Same as I) but across TCA levels. **Fig. S2 related to Fig. 3.** (A) Reduced χ^2^ of PSD pre-stimulus baseline between simulated WT and simulated network with TC and IC glutamatergic synaptic increases. The TC increase was equally imposed on the excitatory and inhibitory neurons (i.e., TCA = 0%). 2WA IC: *p* < 0.001, F = 8.05; TC: *p* < 0.001, F = 10.25; interaction: *p* = 0.01, F = 1.24. (B) Same as A) but imposing the TC increase preferentially to the inhibitory neurons (TCA = 40%). 2WA IC: *p* < 0.001, F = 9.86; TC: *p* < 0.001, F = 8.86; interaction: *p* = 0.81, F = 0.91. (C) Reduced χ^2^ of MUA slope ratio between excitatory and inhibitory neurons when the TC increase was equally imposed on the excitatory and inhibitory neurons (i.e., TCA = 0%). 2WA IC: *p* < 0.001, F = 89.25; TC: *p* < 0.001, F = 7.67; interaction: *p* = 0.99, F = 0.46. (D) Same as C) but imposing the TC increase preferentially to the inhibitory neurons (TCA = 40%). 2WA IC: *p* = 0.14, F = 1.68; TC: *p* < 0.001, F = 85.24; interaction: p = 0.99, F = 0.41. (E) Reduced χ^2^ as a function of IC increase of the ratio between high and low ɣ BB PSD modulation of K = 90 with respect to pre-stimulation with TCA = 0% (blue line) and with TCA = 40% (red line). 2WA IC: *p* < 0.001, F = 5.09; presence/absence of TCA: *p* < 0.001, F = 28.97; interaction: *p* = 0.09, F = 1.84. (F) Same as E) but as a function of TC synapses increase. 2WA TC: *p* = 0.22, F = 1.38; presence/absence of TCA: *p* = 0.003, F = 9.13; interaction: *p* = 0.05, F = 2.1. (G) Recurrent inhibitory over excitatory ratio across visual contrasts in the simulated WT (orange) and FHM1 computational model with TCA = 0% (blue) and TCA = 40% (red). 2WRMA: K: *p* = 0.06, F = 2.89; presence/absence of TCA in FHM1 model (hence not inluding simulated WT I/E ratio): *p* < 0.001, F = 448.76; interaction: *p* = 0.001, F = 7.44. 2WRMA: K: *p* < 0.001, F = 23.24; animal group: *p* < 0.001, F = 106.95; interaction: *p* < 0.001, F = 18.51. (H) Recurrent inhibitory over excitatory ratio across IC and TC increase in the simulated FHM1 computational model when the TC increase was equally imposed to the excitatory and inhibitory neurons (i.e., TCA = 0%). 2WA IC: *p* < 0.001, F = 16.31; TC: *p* < 0.001, F = 59.65. (I) Same as H) but imposing the TC increase preferentially to the inhibitory neurons (TCA = 40%). 2WA IC: *p* < 0.001, F = 92.14; TC: *p* < 0.001, F = 20.38. **Fig. S3 related to Fig. 4.** (A) Schematics of the FHM1 computational model when considering only the thalamocortical (TC) synaptic increase (represented by sand-colored arrows. Please note the arrrow of TC increase targeting inhibitory neurons is larger than the one at excitatory neurons). Note that in the simulations for panels A-D the cortical synaptic increase was set to the level meant for reproducing the FHM1 experimental data as in Fig. 3. (B) PSD modulation of the simulated LFPs at pre-stimulation across TC synaptic increase levels (indictaed in the legend of panel D). The modulation of PSDs was computed with respect to the LFP during pre-stimulation at the TC level adopted for reproducing the experimental data of the FHM1 animal group. (C) PSD modulation of the simulated LFPs at K = 90 across TC synaptic increase levels (indictaed in the legend of panel D). The modulation of PSDs was computed with respect to the LFPs at K = 90 at the TC level adopted for reproducing the experimental data of the FHM1 animal group. (D) PSD modulation of the simulated LFPs at K = 90 wrt baseline across TC synaptic increase levels. (E) Recurrent inhibitory over excitatory ratio across the TC synaptic increase levels. (F) PSD modulation as in (D) but for the low (left), the high (middle), and their ratio (right) broad ɣ band. (G-N) Same as (A-F) but across IC excitatory synaptic increase levels. (O-T) Same as (A-F) but across TCA values.

## Data Availability

The Brian2 simulating environment is available online as open source. Data, custom MATLAB analysis scripts, and Brian2-based Python scripts for running the simulations are available upon reasonable request from the lead contact Alberto Mazzoni (alberto.mazzoni@santannapisa.it).

## Abbreviations

2WA: two-way ANOVA
BB: broad ɣ band
FHM1: familial-hemiplegic-type1-migraine
I/E: inhibitory over excitatory
IC: intra-cortical
K: contrast
L4: layer IV
LFP: local field potential
MUA: multi-unit activity
N1: first negative peak of the visual evoked potential
NB: narrow ɣ band
P2: second positive peak of the visual evoked potential
PSD: power spectral density
STD: short-term depression
std.: standard deviation
TC: thalamo-cortical
TCA: thalamo-cortical asymmetry
V1: primary visual cortex
VEP: visual evoked potential
WT: wild type

## References

[1] Stovner LJ, Hagen K, Linde M, Steiner TJ (2022). The global prevalence of headache: an update, with analysis of the influences of methodological factors on prevalence estimates. J Headache Pain.

[2] Goadsby PJ, Holland PR, Martins-Oliveira M, Hoffmann J, Schankin C, Akerman S (2017). Pathophysiology of migraine: a disorder of sensory processing. Physiol Rev.

[3] Pietrobon D, Moskowitz MA (2013) Pathophysiology of migraine. Annu Rev Physiol 75(1):365–391. 10.1146/annurev-physiol-030212-183717 10.1146/annurev-physiol-030212-183717 23190076

[4] Battista J, Badcock DR, McKendrick AM (2011). Migraine increases Centre-surround suppression for drifting visual stimuli. PLoS One.

[5] Nguyen BN, McKendrick AM, Vingrys AJ (2016). Abnormal inhibition-excitation imbalance in migraine. Cephalalgia.

[6] Vecchia D, Pietrobon D (2012). Migraine: a disorder of brain excitatory–inhibitory balance?. Trends Neurosci.

[7] Aurora S, Wilkinson F (2007). The brain is Hyperexcitable in migraine. Cephalalgia.

[8] Coppola G, Pierelli F, Schoenen J (2007). Is the cerebral cortex Hyperexcitable or Hyperresponsive in migraine?. Cephalalgia.

[9] Cosentino G, Fierro B, Brighina F (2014). From different neurophysiological methods to conflicting pathophysiological views in migraine: a critical review of literature. Clin Neurophysiol.

[10] Pietrobon D, Brennan KC (2019). Genetic mouse models of migraine. J Headache Pain.

[11] Haan J, Kors EE, Vanmolkot KRJ, Maagdenberg AMJM, Frants RR, Ferrari MD (2005). Migraine genetics: an update. Curr Sci Inc.

[12] van den Maagdenberg AMJM (2004). A Cacna1a Knockin migraine mouse model with increased susceptibility to cortical spreading depression. Neuron.

[13] Pietrobon D (2013). Calcium channels and migraine. Biochim Biophys Acta Biomembr.

[14] Tottene A (2002). Familial hemiplegic migraine mutations increase Ca2+ influx through single human CaV2.1 channels and decrease maximal CaV2.1 current density in neurons. Proc Natl Acad Sci.

[15] Tottene A (2009). Enhanced excitatory transmission at cortical synapses as the basis for facilitated spreading depression in CaV2.1 Knockin migraine mice. Neuron.

[16] Tottene A, Favero M, Pietrobon D (2019) Enhanced Thalamocortical Synaptic Transmission and Dysregulation of the Excitatory–Inhibitory Balance at the Thalamocortical Feedforward Inhibitory Microcircuit in a Genetic Mouse Model of Migraine. J Neurosci 39(49):9841–9851. 10.1523/JNEUROSCI.1840-19.2019 10.1523/JNEUROSCI.1840-19.2019 PMC6891058 31645463

[17] Vecchia D, Tottene A, van den Maagdenberg AMJM, Pietrobon D (2014). Mechanism underlying unaltered cortical inhibitory synaptic transmission in contrast with enhanced excitatory transmission in CaV2.1 knockin migraine mice. Neurobiol Dis.

[18] Meneghetti N, Cerri C, Tantillo E, Vannini E, Caleo M, Mazzoni A (2021) Narrow and broad gamma bands process complementary visual information in mouse primary visual cortex. eNeuro ENEURO.0106–21.2021. 10.1523/ENEURO.0106-21.2021 10.1523/ENEURO.0106-21.2021 PMC8570688 34663617

[19] Ko H, Hofer SB, Pichler B, Buchanan KA, Sjöström PJ, Mrsic-Flogel TD (2011). Functional specificity of local synaptic connections in neocortical networks. Nature.

[20] Tantillo E (2020). Differential roles of pyramidal and fast-spiking, GABAergic neurons in the control of glioma cell proliferation. Neurobiol Dis.

[21] Bruyns-Haylett M (2017). The neurogenesis of P1 and N1: a concurrent EEG/LFP study. NeuroImage.

[22] Freeman JA, Nicholson C (1975). Experimental optimization of current source-density technique for anuran cerebellum. J Neurophysiol.

[23] Haberly LB, Shepherd GM (1973). Current-density analysis of summed evoked potentials in opossum prepyriform cortex. J Neurophysiol.

[24] Pettersen KH, Devor A, Ulbert I, Dale AM, Einevoll GT (2006). Current-source density estimation based on inversion of electrostatic forward solution: effects of finite extent of neuronal activity and conductivity discontinuities. J Neurosci Methods.

[25] Shannon CE (1948). A mathematical theory of communication. Bell Syst Tech J.

[26] Magri C, Whittingstall K, Singh V, Logothetis NK, Panzeri S (2009). A toolbox for the fast information analysis of multiple-site LFP, EEG and spike train recordings. BMC Neurosci.

[27] Panzeri S, Treves A (1996). Analytical estimates of limited sampling biases in different information measures. Netw Comput Neural Syst.

[28] Cohen MX (2014). Analyzing neural time series data: theory and practice.

[29] Cavallari S, Panzeri S, Mazzoni A (2014) Comparison of the dynamics of neural interactions between current-based and conductance-based integrate-and-fire recurrent networks. Front Neural Circuits 8. 10.3389/fncir.2014.00012 10.3389/fncir.2014.00012 PMC3943173 24634645

[30] Mazzoni A, Panzeri S, Logothetis NK, Brunel N (2008). Encoding of naturalistic stimuli by local field potential spectra in networks of excitatory and inhibitory neurons. PLoS Comput Biol.

[31] Mazzoni A, Whittingstall K, Brunel N, Logothetis NK, Panzeri S (2010). Understanding the relationships between spike rate and delta/gamma frequency bands of LFPs and EEGs using a local cortical network model. NeuroImage.

[32] Mazzoni A, Brunel N, Cavallari S, Logothetis NK, Panzeri S (2011). Cortical dynamics during naturalistic sensory stimulations: experiments and models. J Physiol-Paris.

[33] Tuckwell HC (1988). Introduction to theoretical neurobiology.

[34] Braitenberg V, Schüz A (1991) Anatomy of the cortex, 18. Berlin: Springer Berlin Heidelberg. 10.1007/978-3-662-02728-8

[35] Holmgren C, Harkany T, Svennenfors B, Zilberter Y (2003). Pyramidal cell communication within local networks in layer 2/3 of rat neocortex. J Physiol.

[36] Sjöström PJ, Turrigiano GG, Nelson SB (2001). Rate, timing, and cooperativity jointly determine cortical synaptic plasticity. Neuron.

[37] Brunel N, Wang X-J (2003). What determines the frequency of fast network oscillations with irregular neural discharges? I. synaptic dynamics and excitation-inhibition balance. J Neurophysiol.

[38] Bartos M, Vida I, Frotscher M, Geiger JRP, Jonas P (2001). Rapid signaling at inhibitory synapses in a dentate gyrus interneuron network. J Neurosci.

[39] Bartos M (2002). Fast synaptic inhibition promotes synchronized gamma oscillations in hippocampal interneuron networks. Proc Natl Acad Sci.

[40] Gupta A (2000). Organizing principles for a diversity of GABAergic interneurons and synapses in the neocortex. Science.

[41] Markram H, Lübke J, Frotscher M, Roth A, Sakmann B (1997). Physiology and anatomy of synaptic connections between thick tufted pyramidal neurones in the developing rat neocortex. J Physiol.

[42] Angulo MC, Rossier J, Audinat E (1999). Postsynaptic glutamate receptors and integrative properties of fast-spiking interneurons in the rat neocortex. J Neurophysiol.

[43] Kraushaar U, Jonas P (2000). Efficacy and stability of quantal GABA release at a hippocampal interneuron-principal neuron synapse. J Neurosci.

[44] Xiang Z, Huguenard JR, Prince DA (1998). GABA _A_ receptor-mediated currents in interneurons and pyramidal cells of rat visual cortex. J Physiol.

[45] Zhou F-M, Hablitz JJ (1998). AMPA receptor-mediated EPSCs in rat neocortical layer II/III interneurons have rapid kinetics. Brain Res.

[46] Saleem AB (2017). Subcortical source and modulation of the narrowband gamma oscillation in mouse visual cortex. Neuron.

[47] Mazzoni A, Lindén H, Cuntz H, Lansner A, Panzeri S, Einevoll GT (2015). Computing the local field potential (LFP) from integrate-and-fire network models. PLoS Comput Biol.

[48] Hansel D, Mato G, Meunier C, Neltner L (1998). On numerical simulations of integrate-and-fire neural networks. Neural Comput.

[49] Shelley MJ, Tao L (2001). Efficient and accurate time-stepping schemes for integrate-and-fire neuronal networks. J Comput Neurosci.

[50] Goodman D (2008) Brian: a simulator for spiking neural networks in Python. Front Neuroinform 2. 10.3389/neuro.11.005.2008 10.3389/neuro.11.005.2008 PMC2605403 19115011

[51] Stimberg M, Brette R, Goodman DF (2019) Brian 2, an intuitive and efficient neural simulator. eLife 8:e47314. 10.7554/eLife.47314 10.7554/eLife.47314 PMC6786860 31429824

[52] Theiler J, Eubank S, Longtin A, Galdrikian B, Doyne Farmer J (1992). Testing for nonlinearity in time series: the method of surrogate data. Physica D: Nonlinear Phenomena.

[53] Ji X, Zingg B, Mesik L, Xiao Z, Zhang LI, Tao HW (2016). Thalamocortical innervation pattern in mouse auditory and visual cortex: laminar and cell-type specificity. Cereb Cortex.

[54] Di Russo F, Martínez A, Sereno MI, Pitzalis S, Hillyard SA (2002). Cortical sources of the early components of the visual evoked potential: cortical sources of VEP. Hum Brain Mapp.

[55] Liao W, Gandal MJ, Ehrlichman RS, Siegel SJ, Carlson GC (2012). MeCP2+/− mouse model of RTT reproduces auditory phenotypes associated with Rett syndrome and replicate select EEG endophenotypes of autism spectrum disorder. Neurobiol Dis.

[56] Ridder WH, Nusinowitz S (2006). The visual evoked potential in the mouse—origins and response characteristics. Vis Res.

[57] Timme NM, C. Lapish (2018) A Tutorial for Information Theory in Neuroscience eNeuro 5:3. ENEURO.0052–18.2018. 10.1523/ENEURO.0052-18.2018 10.1523/ENEURO.0052-18.2018 PMC6131830 30211307

[58] Adesnik H (2017). Synaptic mechanisms of feature coding in the visual cortex of awake mice. Neuron.

[59] Brennan KC, Pietrobon D (2018). A systems neuroscience approach to migraine. Neuron.

[60] Younis S, Hougaard A, Noseda R, Ashina M (2019). Current understanding of thalamic structure and function in migraine. Cephalalgia.

[61] Gielen S, Krupa M, Zeitler M (2010). Gamma oscillations as a mechanism for selective information transmission. Biol Cybern.

[62] Harris KD, Csicsvari J, Hirase H, Dragoi G, Buzsáki G (2003). Organization of cell assemblies in the hippocampus. Nature.

[63] Vick M, Womelsdorf T, Fries, Pascal (2013) Gamma-Band Synchronization and Information Transmission. in Principles of Neural Coding. Boca Raton: CRC Press. 449–462

[64] Pritchett DL, Siegle JH, Deister CA, Moore CI (2015). For things needing your attention: the role of neocortical gamma in sensory perception. Curr Opin Neurobiol.

[65] Mulleners WM, Chronicle EP, Palmer JE, Koehler PJ, Vredeveld J-W (2001). Visual cortex excitability in migraine with and without Aura. Headache.

[66] Puledda F, Ffytche D, O’Daly O, Goadsby PJ (2019). Imaging the visual network in the migraine Spectrum. Front Neurol.

[67] Pietrobon D, Moskowitz MA (2014). Chaos and commotion in the wake of cortical spreading depression and spreading depolarizations. Nat Rev Neurosci.

[68] Parker PD (2021). Non-canonical glutamate signaling in a genetic model of migraine with aura. Neuron.

[69] Crivellaro G (2021). Specific activation of GluN1-N2B NMDA receptors underlies facilitation of cortical spreading depression in a genetic mouse model of migraine with reduced astrocytic glutamate clearance. Neurobiol Dis.

[70] Henrie JA, Shapley R (2005). LFP power spectra in V1 cortex: the graded effect of stimulus contrast. J Neurophysiol.

[71] Brunel N, Hakim V (1999). Fast global oscillations in networks of integrate-and-fire neurons with low firing rates. Neural Comput.

[72] Perenboom MJL, Schenke M, Ferrari MD, Terwindt GM, den Maagdenberg AMJM, Tolner EA (2021). Responsivity to light in familial hemiplegic migraine type 1 mutant mice reveals frequency-dependent enhancement of visual network excitability. Eur J Neurosci.

[73] Han C, Shapley R, Xing D (2021) Gamma rhythms in the visual cortex: functions and mechanisms. Cogn Neurodyn. 10.1007/s11571-021-09767-x 10.1007/s11571-021-09767-x PMC9279528 35847544

[74] Niell CM, Stryker MP (2010). Modulation of visual responses by behavioral state in mouse visual cortex. Neuron.

[75] Belitski A (2008). Low-frequency local field potentials and spikes in primary visual cortex convey independent visual information. J Neurosci.

[76] Pietrobon D (2018). Ion channels in migraine disorders. Curr Opin Physiol.

[77] Tremblay R, Lee S, Rudy B (2016). GABAergic interneurons in the neocortex: from cellular properties to circuits. Neuron.

[78] Marchionni I, Pilati N, Forli A, Sessolo M, Tottene A, Pietrobon D (2022) Enhanced Feedback Inhibition Due to Increased Recruitment of Somatostatin-Expressing Interneurons and Enhanced Cortical Recurrent Excitation in a Genetic Mouse Model of Migraine. J Neurosci JN-RM-0228-22. 10.1523/JNEUROSCI.0228-22.2022 10.1523/JNEUROSCI.0228-22.2022 PMC9410751 35863891

[79] Billeh YN et al. (2020) Systematic Integration of Structural and Functional Data into Multi-scale Models of Mouse Primary Visual Cortex. Neuron S0896627320300672. 10.1016/j.neuron.2020.01.040 10.1016/j.neuron.2020.01.040 32142648

[80] Campagnola L et al. (2022) Local connectivity and synaptic dynamics in mouse and human neocortex. Science 375(6585):eabj5861. 10.1126/science.abj5861 10.1126/science.abj5861 PMC9970277 35271334

