# Supplementary material for: Synaptic alterations in visual cortex reshape contrast-dependent gamma oscillations and inhibition-excitation ratio in a genetic mouse model of migraine
Source: J Headache Pain. 2022 Sep 29;23(1):125. doi: 10.1186/s10194-022-01495-9 (PMC9523950; doi:10.1186/s10194-022-01495-9)
Supplement: Supplementary file 1 — Fig. S1 related to Figs. 3, 4. (A) (left) PSD modulation for the minimum contrast level (K = 0) of experimental (black) and simulated data for WT (orange, left) and FHM1 (green, right). ${\chi}_r^2=0.34$χr2=0.34 for WT and ${\chi}_r^2=0.28$χr2=0.28 for FHM1. (B) Cortical narrow band power modulation as a function of thalamic narrow band strength in the FHM1 model. The model was set with (red) and without (dark blue) waning of FHM1 thalamocortical strengthening at the high rate of LGN neurons generating NB (mean ± SEM). This phenomenon was simulated by injecting the thalamic NB in thalamocortical synapses with (blue) or without (red) the gain-of-function induced by the FHM1 mutations. 2WRMA: Thalamic NB: p < 0.001, F = 1666; presence/absence of TC increase: p < 0.001, F = 157.45; interaction: p < 0.001, F = 43.28. (C) Excitatory (dashed black line) and inhibitory (black line) simulated firing rate across TC increase levels (mean ± std). (D) Same as C) but across IC synaptic increase levels. (E) Same as C) but across TCA. (F) Inhibitory over excitatory ratio (mean ± std) in the simulated excitatory neurons across TC increase levels (in the legend). Please note the difference with respect to the recurrent I/E ratio of Fig. 3D, 4CHO (See Methods). (G) Same as F) but across IC increase levels (indicated in the legend). (H) Same as F) but across TCA levels (indicated in the legend). (I) Recurrent inhibitory over excitatory ratio (mean ± std) in the simulated excitatory neurons across TC increa [file 10194_2022_1495_MOESM1_ESM.docx]

**
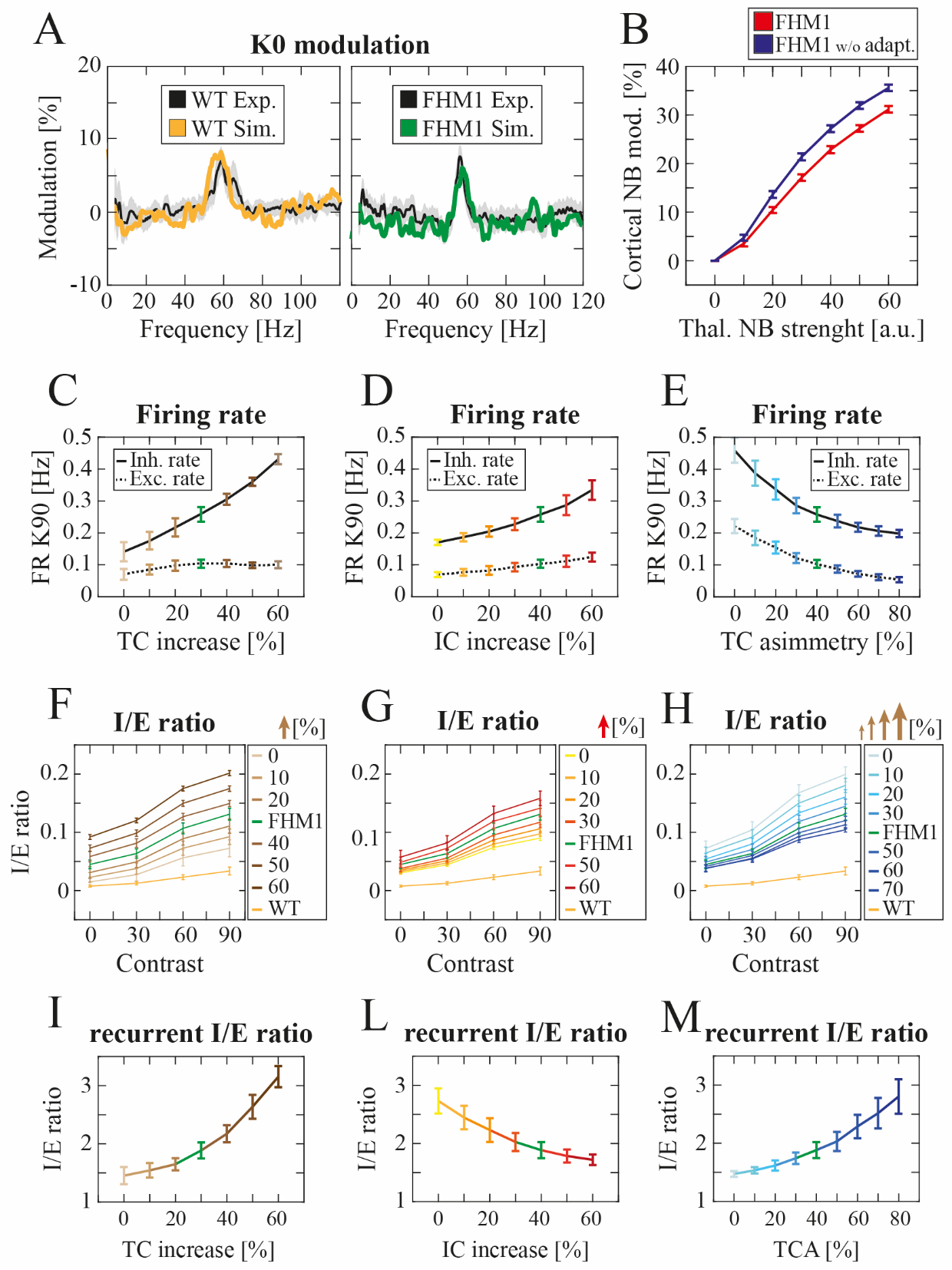
Supplementary figures**

**Figure S1 related to Figure 3,4.**

(A) (left) PSD modulation for the minimum contrast level (K=0) of experimental (black) and simulated data for WT (orange, left) and FHM1 (green, right). $\chi_{r}^{2}=0.34$ for WT and $\chi_{r}^{2}=0.28$ for FHM1.

(B) Cortical narrow band power modulation as a function of thalamic narrow band strength in the FHM1 model. The model was set with (red) and without (dark blue) waning of FHM1 thalamocortical strengthening at the high rate of LGN neurons generating NB (mean ± SEM). This phenomenon was simulated by injecting the thalamic NB in thalamocortical synapses with (blue) or without (red) the gain-of-function induced by the FHM1 mutations. 2WRMA: Thalamic NB: p<0.001, F=1666; presence/absence of TC increase: p<0.001, F=157.45; interaction: p<0.001, F=43.28.

(C) Excitatory (dashed black line) and inhibitory (black line) simulated firing rate across TC increase levels (mean ± std)

(D) Same as C) but across IC synaptic increase levels.

(E) Same as C) but across TCA.

(F) Inhibitory over excitatory ratio (mean ± std) in the simulated excitatory neurons across TC increase levels (in the legend). Please note the difference with respect to the recurrent I/E ratio of Figures 3D, 4CHO (See Methods).

(G) Same as F) but across IC increase levels (indicated in the legend).

(H) Same as F) but across TCA levels (indicated in the legend).

(I) Recurrent inhibitory over excitatory ratio (mean ± std) in the simulated excitatory neurons across TC increase levels (in the legend of panel F).

(L) Same as I) but across IC increase levels.

(M) Same as I) but across TCA levels.


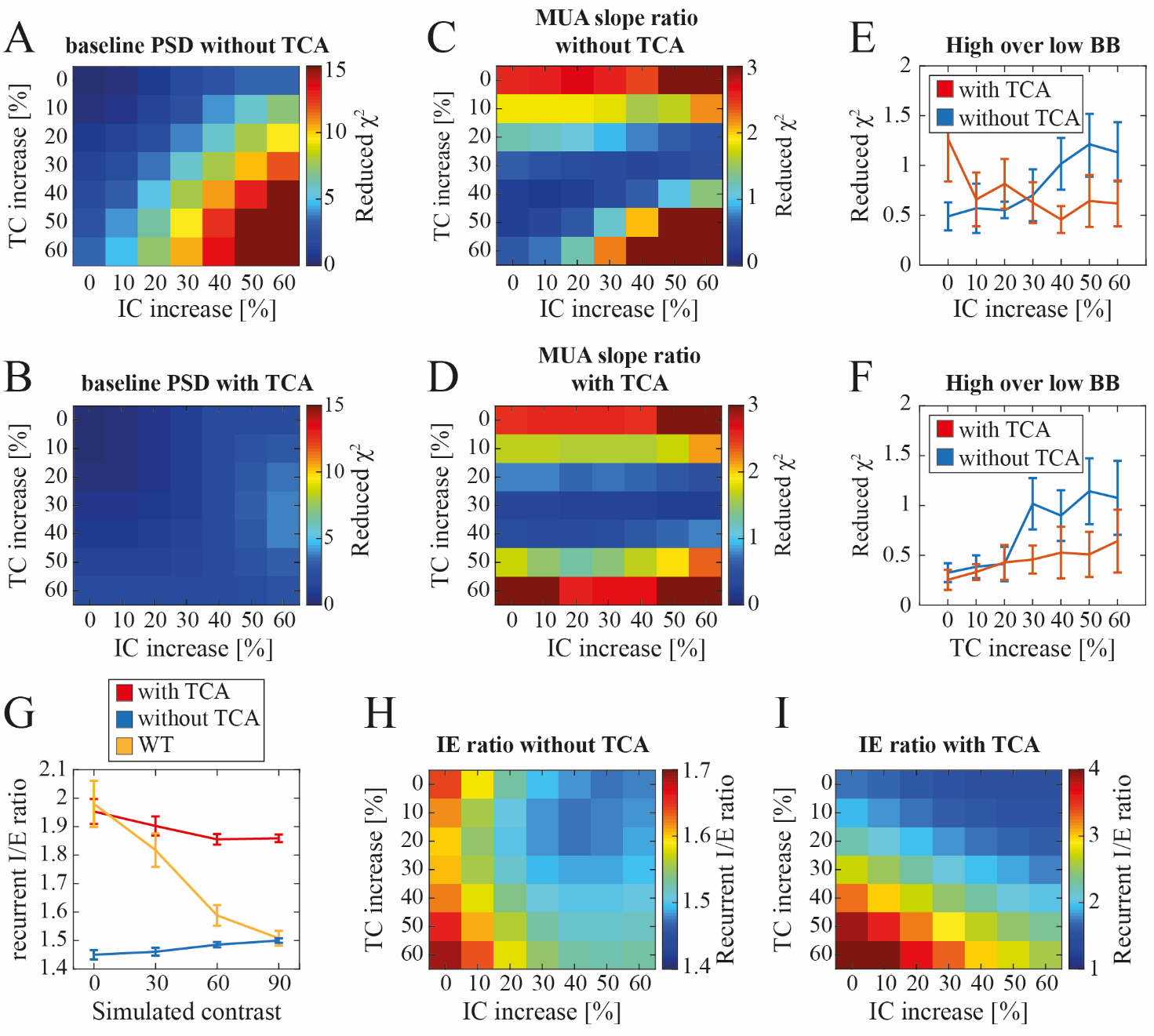


**Figure S2 related to Figure 3.**

(A) Reduced χ^2^ of PSD pre-stimulus baseline between simulated WT and simulated network with TC and IC glutamatergic synaptic increases. The TC increase was equally imposed on the excitatory and inhibitory neurons (i.e., TCA=0%). 2WA IC: p<0.001, F=8.05; TC: p<0.001, F=10.25; interaction: p=0.01, F=1.24.

(B) Same as A) but imposing the TC increase preferentially to the inhibitory neurons (TCA=40%). 2WA IC: p<0.001, F=9.86; TC: p<0.001, F=8.86; interaction: p=0.81, F=0.91.

(C) Reduced χ^2^ of MUA slope ratio between excitatory and inhibitory neurons when the TC increase was equally imposed to the excitatory and inhibitory neurons (i.e., TCA=0%). 2WA IC: p<0.001, F=89.25; TC: p<0.001, F=7.67; interaction: p=0.99, F=0.46.

(D) Same as C) but imposing the TC increase preferentially to the inhibitory neurons (TCA=40%). 2WA IC: p=0.14, F=1.68; TC: p<0.001, F=85.24; interaction: p=0.99, F=0.41.

(E) Reduced χ^2^ as a function of IC increase of the ratio between high and low ɣ BB PSD modulation of K=90 with respect to pre-stimulation with TCA=0% (blue line) and with TCA=40% (red line). 2WA IC: p<0.001, F=5.09; presence/absence of TCA: p<0.001, F=28.97; interaction: p=0.09, F=1.84.

(F) Same as E) but as a function of TC synapses increase. 2WA TC: p=0.22, F=1.38; presence/absence of TCA: p=0.003, F=9.13; interaction: p=0.05, F=2.1.

(G) Recurrent inhibitory over excitatory ratio across visual contrasts in the simulated WT (orange) and FHM1 computational model with TCA=0% (blue) and TCA=40% (red). 2WRMA: K: p=0.06, F=2.89; presence/absence of TCA in FHM1 model (hence not inluding simulated WT I/E ratio): p<0.001, F=448.76; interaction: p=0.001, F=7.44. 2WRMA: K: p<0.001, F=23.24; animal group: p<0.001, F=106.95; interaction: p<0.001, F=18.51.

(H) Recurrent inhibitory over excitatory ratio across IC and TC increase in the simulated FHM1 computational model when the TC increase was equally imposed to the excitatory and inhibitory neurons (i.e., TCA=0%). 2WA IC: p<0.001, F=16.31; TC: p<0.001, F=59.65.

(I) Same as H) but imposing the TC increase preferentially to the inhibitory neurons (TCA=40%). 2WA IC: p<0.001, F=92.14; TC: p<0.001, F=20.38.


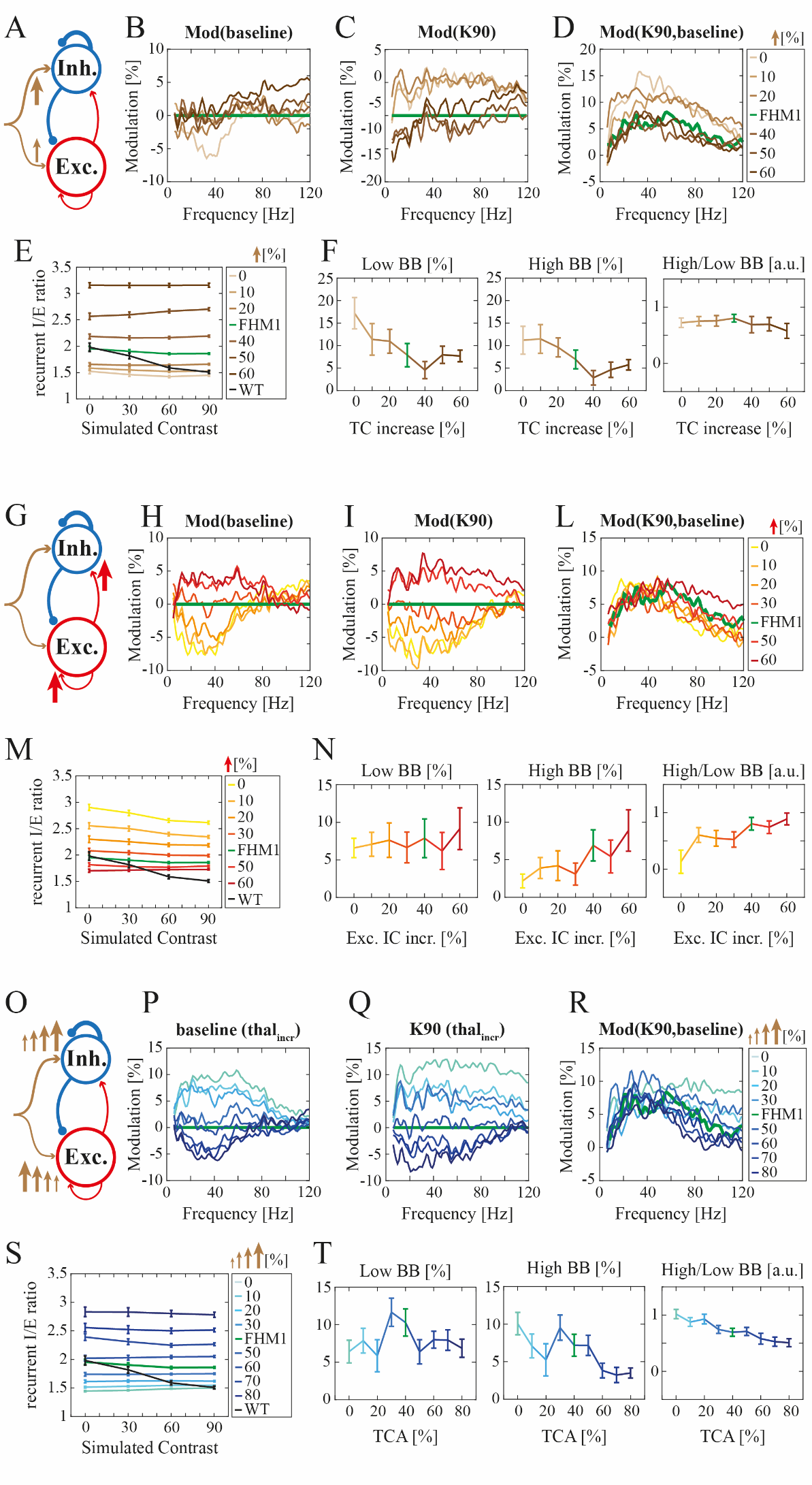


**Figure S3 related to Figure 4.**

(A) Schematics of the FHM1 computational model when considering only the thalamocortical (TC) synaptic increase (represented by sand-colored arrows. Please note the arrrow of TC increase targeting inhibitory neurons is larger than the one at excitatory neurons). Note that in the simulations for panels A-D the cortical synaptic increase was set to the level meant for reproducing the FHM1 experimental data as in Figure 3.

(B) PSD modulation of the simulated LFPs at pre-stimulation across TC synaptic increase levels (indictaed in the legend of panel D). The modulation of PSDs was computed with respect to the LFP during pre-stimulation at the TC level adopted for reproducing the experimental data of the FHM1 animal group.

(C) PSD modulation of the simulated LFPs at K=90 across TC synaptic increase levels (indictaed in the legend of panel D). The modulation of PSDs was computed with respect to the LFPs at K=90 at the TC level adopted for reproducing the experimental data of the FHM1 animal group.

(D) PSD modulation of the simulated LFPs at K=90 wrt baseline across TC synaptic increase levels.

(E) Recurrent inhibitory over excitatory ratio across the TC synaptic increase levels.

(F) PSD modulation as in (D) but for the low (left), the high (middle), and their ratio (right) broad ɣ band.

(G-N) Same as (A-F) but across IC excitatory synaptic increase levels.

(O-T) Same as (A-F) but across TCA values.
